# Teasing apart retrieval and encoding interference in the processing of anaphors

**DOI:** 10.3389/fpsyg.2015.00506

**Published:** 2015-06-09

**Authors:** Lena A. Jäger, Lena Benz, Jens Roeser, Brian W. Dillon, Shravan Vasishth

**Affiliations:** ^1^Department of Linguistics, University of PotsdamPotsdam, Germany; ^2^Department of Psychology, Nottingham Trent UniversityNottingham, UK; ^3^Department of Linguistics, University of MassachusettsAmherst, MA, USA

**Keywords:** anaphors, reflexives, possessives, eye-tracking, German, Swedish, working-memory, interference

## Abstract

Two classes of account have been proposed to explain the memory processes subserving the processing of reflexive-antecedent dependencies. Structure-based accounts assume that the retrieval of the antecedent is guided by syntactic tree-configurational information without considering other kinds of information such as gender marking in the case of English reflexives. By contrast, unconstrained cue-based retrieval assumes that all available information is used for retrieving the antecedent. Similarity-based interference effects from structurally illicit distractors which match a non-structural retrieval cue have been interpreted as evidence favoring the unconstrained cue-based retrieval account since cue-based retrieval interference from structurally illicit distractors is incompatible with the structure-based account. However, it has been argued that the observed effects do not necessarily reflect interference occurring at the moment of retrieval but might equally well be accounted for by interference occurring already at the stage of encoding or maintaining the antecedent in memory, in which case they cannot be taken as evidence against the structure-based account. We present three experiments (self-paced reading and eye-tracking) on German reflexives and Swedish reflexive and pronominal possessives in which we pit the predictions of encoding interference and cue-based retrieval interference against each other. We could not find any indication that encoding interference affects the processing ease of the reflexive-antecedent dependency formation. Thus, there is no evidence that encoding interference might be the explanation for the interference effects observed in previous work. We therefore conclude that invoking encoding interference may not be a plausible way to reconcile interference effects with a structure-based account of reflexive processing.

## 1. Introduction

A central task the human sentence processing mechanism has to accomplish is to link two parts of a syntactic dependency, irrespective of how much linguistic material separates the two dependents. Many theories of sentence processing therefore assume that upon encountering the second dependent, the parser triggers a memory retrieval to access the first dependent in order to integrate it with the current node (Gibson, 2000; Lewis and Vasishth, 2005). Interference effects have recently come into focus in sentence processing research because they are taken to be informative about the more precise nature of the retrieval mechanisms that subserve sentence processing. However, the relationship between empirically observed similarity-based interference effects and theories of retrieval is somewhat indirect, because there are multiple distinct mechanisms that could give rise to similarity-based interference effects in online processing. Indeed, whether or not the observation of interference effects can be interpreted as evidence favoring one or another account of sentence processing depends on the exact mechanisms causing the interference effects. In this article, we will present different mechanisms that have been proposed to account for interference effects in sentence comprehension and present three experiments with different methodologies and languages to tease them apart. We will first give an overview of two kinds of mechanisms, *cue-based retrieval interference* and *encoding interference*, which in the working memory literature have been proposed to underly similarity-based interference. Subsequently, we will turn to the implications for sentence processing and antecedent-retrieval in the processing of reflexives in particular.

Similarity-based interference has long been known to be a major cause of forgetting (Anderson and Neely, 1996). In memory models which represent items as bundles or vectors of features, similarity-based interference is assumed to arise as a function of the degree of overlap between an item's features with the features of other items in memory (Nairne, 1988, 1990; Anderson and Neely, 1996; Anderson and Lebiere, 1998; Anderson et al., 2004; Oberauer and Kliegl, 2006; Lewandowsky et al., 2008). However, the various memory models differ with respect to the mechanisms which they assume to underlie similarity-based interference. Generally speaking, one can distinguish between two kinds of similarity-based interference. On the one hand, similarity-based interference is assumed to affect the encoding or maintenance of an item (Nairne, 1988, 1990; Oberauer and Kliegl, 2006; Lewandowsky et al., 2008). We will refer to this proposal as *encoding interference*. On the other hand, similarity-based interference is assumed to arise during the retrieval of an item (Anderson and Neely, 1996; Anderson and Lebiere, 1998; Anderson et al., 1998, 2004; McElree, 2006; Oberauer and Kliegl, 2006). We will refer to this second proposal as *cue-based retrieval interference*.

Encoding interference is assumed to arise from the competition between the features of similar items that occurs at the moment of encoding or maintaining items in memory. Nairne (1990), for instance, proposed that whenever two items share a feature, they compete for this feature. In a certain proportion of cases, the memory representation of one of these items therefore loses this feature.^1^ Hence, this item's memory representation becomes less distinct from other items and, as a result, retrieval probability decreases. An important, but subtle, point here is that even though encoding interference arises at the stage of encoding or maintaining an item in memory, it has an impact on the ease of this item's later *retrieval*. Oberauer and Kliegl (2006), who adopted Nairne (1990)'s concept of feature-overwriting, implemented the idea of an item's memory representation being degraded by decreasing this item's activation level. At the moment of later retrieval, this lower activation level leads to lower retrieval probability and a slow-down in processing times. In their model, the retrieval of an item from working memory is implemented as its gradual activation into the focus layer of the memory system. The processing speed of this gradual activation is defined as a function of this item's activation level prior to retrieval. Thus, if an item's activation level is decreased due to encoding interference from competitor items, a slow-down in the retrieval process is predicted. Note that Oberauer and Kliegl (2006) do not make any predictions about retrieval latencies. Their model is designed to explain data collected in speed-accuracy tradeoff experiments, where they experimentally controlled the time point when retrieval was supposed to happen. In their model, the slow-down in the retrieval process therefore is reflected in a higher proportion of retrieval failures rather than in increased retrieval latencies because participants are forced to interrupt the retrieval process after an experimentally defined time lag. Translating the Oberauer and Kliegl (2006) model to sentence processing, where the participant has more time to carry out retrieval, leads us to the assumption that the slow-down in the retrieval process is reflected in longer retrieval latencies. For the predictions of the experiments reported in this article, we will refer to encoding interference as implemented in the Oberauer and Kliegl (2006) model, with the additional assumption that a slow-down in the retrieval process leads to increased retrieval latencies. In sum, although encoding interference acts at the moment of encoding and maintenance rather than at retrieval, it indirectly affects the success and the speed of the retrieval process because it results in a representation that is more difficult to access.

Cue-based retrieval interference, in contrast, is assumed to arise due to cue-overload at the moment of retrieval. In a content-addressable memory architecture, cue-overload refers to a scenario when the cues used for retrieval do not point to a unique target, but rather match multiple items (Watkins and Watkins, 1975). This is assumed to lead to misretrievals of partially matching distractor items (Anderson and Lebiere, 1998; Anderson et al., 2004; McElree, 2006) and mutual inhibition between the distractors and the target resulting in a higher retrieval latency in case the target and the distractor have one or more retrieval relevant features in common (Anderson and Lebiere, 1998; Anderson et al., 2004).^2^ To summarize, encoding interference is predicted to occur whenever items share features, no matter whether these features are used for retrieval or not. Cue-based retrieval interference, in contrast, is predicted to occur when more than one item matches the retrieval features. Inhibition between these items occurs only when they match the same retrieval features, otherwise cue-based retrieval interference is reflected only in misretrievals (Anderson et al., 2004). Note that encoding interference and cue-based retrieval interference are not mutually exclusive concepts. Indeed, in Oberauer and Kliegl (2006)'s working memory model, both retrieval and encoding interference are assumed and the authors show that their interference model is indeed able to account for a large range of data.

In sentence processing research, early studies investigating interference effects point rather toward encoding than cue-based retrieval interference, but they were not designed to disentangle the two. For example, Gordon et al. (2002) conducted a self-paced reading experiment where participants held a set of nouns in memory while reading the target sentence. The authors report a slow-down in reading times when the noun type (common noun vs. proper name) of the memory load words matched the nouns in the sentence compared to when the memory load nouns and the nouns in the sentence were of different types. These results are further supported by Fedorenko et al. (2006), who also observed similarity-based interference in a memory-load paradigm. Gordon and colleagues report similar results for studies that manipulated similarity between sentence internal nouns rather than memory load (Gordon et al., 2001, 2004, 2006). An example item taken from Gordon et al. (2006) is shown in (1).

**Table:** 

(1)	**Interference/No interference**
	***The banker** that **the barber/Sophie** praised climbed the mountain …*

Since in all of these studies, similarity of the nouns was manipulated while the efficiency of the retrieval cues (i.e., the degree to which the retrieval cues uniquely identify the target) remained constant across experimental conditions, the data reported by Gordon and colleagues favor rather encoding than cue-based retrieval interference as an explanation. However, as Van Dyke and McElree (2006) noted, the above cited studies found interference effects only in the region where the critical noun phrase was retrieved (i.e., at the region containing the verb). This might indicate that the observed effect should rather be attributed to cue-based retrieval interference since encoding interference should also affect processing ease at the moment of encoding, i.e., at the moment when the second of the similar nouns is first being encountered. Van Dyke and McElree (2006) conducted a memory load experiment where, in contrast to the memory load experiments reported by Gordon et al. (2002) and Fedorenko et al. (2006), the memory load words were held constant across experimental conditions, but the retrieval cues at the verb were manipulated. The experimental items consisted of object-cleft sentences in which the main clause object preceded the main clause verb (the critical region where retrieval was triggered); for an example taken from Van Dyke and McElree (2006) see (2).

**Table:** 

(2)	**Interference/No interference**
	*It was the boat that the guy who lived by the sea **sailed/ fixed** in two sunny days.*
	Memory load: *table*, *sink*, *truck*

When the memory load words fit the semantic constraints of the verb, a slow-down in self-paced reading times was observed. These results cannot be attributed to encoding interference since the degree of similarity between the memory load words and the verb's object NP is constant across conditions. Van Dyke and McElree (2006)'s data are thus clear evidence for cue-based retrieval interference playing a role in sentence processing. However, note that the possibility that both retrieval and encoding interference affect sentence processing ease cannot be excluded by Van Dyke and McElree (2006)'s study since their data is clear evidence for cue-based retrieval interference but no evidence *against* encoding interference affecting sentence processing in general.

In recent years, interference effects in the processing of reflexive-antecedent dependencies have drawn considerable attention. The underlying research question was whether unconstrained cue-based retrieval, as proposed by Badecker and Straub (2002) and Patil, Vasishth, and Lewis (unpublished manuscript), or a structure-based access mechanism, as proposed by Nicol and Swinney (1989) and Sturt (2003), subserves the processing of reflexive-antecedent dependencies. Unconstrained cue-based retrieval assumes that all available cues are used to retrieve a reflexive's antecedent. Structure-based accounts, in contrast, assume that structural, i.e., syntactic tree-configurational, information guides the retrieval process. Interference effects in reflexive processing have been generally interpreted in terms of cue-based retrieval interference and taken as evidence for a cue-based retrieval mechanism since *retrieval* interference from syntactically inaccessible constituents is incompatible with the structure-based account. However, as pointed out by Dillon (2011) and Dillon et al. (2013), many of the observed effects—which we will describe more in detail below—can equally well be accounted for by *encoding* interference and hence are not necessarily incompatible with the structure-based account. Indeed, for the kind of materials commonly used to investigate the processing of reflexives (see 3; example taken from Sturt, 2003), encoding interference makes the same predictions for all experimental conditions as the unconstrained cue-based retrieval account implemented in the Lewis and Vasishth (2005) sentence processing model, which is based on the general cue-based architecture Adaptive Control of Thought-Rational (ACT-R) (Anderson and Lebiere, 1998; Anderson et al., 2004) and has been widely used for modeling the processing of reflexives (Dillon, 2011; Dillon et al., 2013; Parker and Phillips, 2014; Kush and Phillips, 2014; Jäger et al., 2015; Patil et al., unpublished manuscript).^3^ Thus, for the question of structure-based vs. unconstrained cue-based retrieval in reflexives, it is crucial to disentangle encoding from cue-based retrieval interference. If evidence can be found showing that encoding interference plays a role in the type of materials generally used to investigate the processing of reflexives, this implies that the interference effects that have been interpreted as evidence favoring unconstrained cue-based retrieval are equally well compatible with a structure-based account.

**Table:** 

(3)	
a.	**Antecedent-match; distractor-match**
	The **surgeon_*i*_** who treated **Jonathan_*j*_** had pricked **himself**_i/^*^j_ …
b.	**Antecedent-match; distractor-mismatch**
	The **surgeon_*i*_** who treated **Jennifer_*j*_** had pricked **himself**_i/^*^j_ …
c.	**Antecedent-mismatch; distractor-match**
	The **surgeon_*i*_** who treated **Jennifer_*j*_** had pricked **herself**_i/^*^j_ …
d.	**Antecedent-mismatch; distractor-mismatch**
	The **surgeon_*i*_** who treated **Jonathan_*j*_** had pricked **herself**_i/^*^j_ …

Studies investigating interference effects in the processing of reflexives mostly tested sentences in which the reflexive was bound by the local subject which c-commanded the reflexive (*surgeon* in Example 3; henceforth referred to as *antecedent*). We will express the antecedent's conformance to the structural requirements for binding the reflexive by attributing the feature {c-com:+} to it.^4^ The interference manipulation was achieved by inserting another noun phrase in a structurally inaccessible position, i.e., not c-commanding the reflexive ({c-com:-}) and hence not qualifying as a binder for the reflexive (*Jonathan/Jennifer* in Example 3; henceforth referred to as *distractor*). A non-structural feature (e.g., gender or number in English reflexives) of this distractor was manipulated. Crucially, the feature which was manipulated might theoretically be used as a retrieval cue. For example, in the processing of English reflexives, the gender feature {gender:masc/fem} marked at the reflexive *himself* or *herself* might be used as a cue to retrieve the antecedent. Thus, if gender is used as a retrieval cue, a gender-matching distractor is predicted to cause cue-based retrieval interference as compared to a distractor which does not match the gender of the reflexive. Therefore, interference effects caused by a feature-matching distractor can be interpreted as evidence favoring an unconstrained cue-based retrieval account. If, in contrast, no effect of a feature-matching distractor is observed, this can be taken as evidence for a structure-based account. This experimental design (or a variation thereof) was used by a large number of studies which aimed to decide whether an unconstrained cue-based retrieval or a structure-based access underlies the processing of reflexive antecedent-dependencies (Nicol and Swinney, 1989; Badecker and Straub, 2002; Sturt, 2003; Xiang et al., 2009; Chen et al., 2012; King et al., 2012; Cunnings and Felser, 2013; Dillon et al., 2013; Clackson and Heyer, 2014; Kush and Phillips, 2014; Parker and Phillips, 2014; Jäger et al., 2015; Patil et al., unpublished manuscript). Some of the cited studies also manipulated feature-match of the structurally accessible antecedent (*surgeon* in Example 3).^5^ An effect of antecedent match/mismatch can be accounted for by both unconstrained cue-based retrieval and structure-based accounts.

The results of the above cited studies are mixed. In antecedent-match conditions, increased processing difficulty due to the presence of a cue-matching distractor has been reported by Badecker and Straub (2002), Experiments 3, 4, Chen et al. (2012), Clackson and Heyer (2014), Jäger et al. (2015), Experiment 2, and Patil et al. (unpublished manuscript). By contrast, Sturt (2003), Experiment 1, and Cunnings and Felser (2013), Experiment 2 found a facilitation due to a cue-matching distractor. It should be noted that in (Sturt, 2003)'s experiment, the effect appeared only delayed and in Cunnings and Felser (2013)'s study, the interference effect was only observed in participants with low working-memory span. Importantly, in a large number of studies, no interference effect in antecedent-match conditions was observed (Nicol and Swinney, 1989; Badecker and Straub, 2002, Experiments 5, 6; Sturt, 2003, Experiment 2; King et al., 2012; Dillon et al., 2013; Kush and Phillips, 2014; Parker and Phillips, 2014; Jäger et al., 2015, Experiment 1). In antecedent-mismatch conditions, a significant processing speed-up due to a cue-matching distractor is reported by King et al. (2012) and Parker and Phillips (2014). The opposite direction of the effect was only observed in Jäger et al. (2015), Experiment 1. The absence of an effect in antecedent-mismatch conditions is reported by Sturt (2003), Xiang et al. (2009) and Dillon et al. (2013). For a literature review of interference effects in reflexives, see Jäger et al. (2015).

As mentioned above, unconstrained cue-based retrieval as implemented in the Lewis and Vasishth (2005) ACT-R model of sentence processing makes precisely the same predictions as encoding interference for sentences like the ones shown in (3). For conditions with a cue-matching antecedent (see 3a,b), the Lewis and Vasishth (2005) model predicts cue-based retrieval interference when the distractor matches the gender of the reflexive (3a). This retrieval interference is predicted to be reflected in inhibition between the antecedent and the distractor because in (3a), but not in (3b), the antecedent (*surgeon*) and the distractor (*Jonathan*) share the gender cue {gender:masc}. Thus, longer retrieval latencies (and hence longer reading times at the reflexive) are predicted in (3a) compared to (3b). Moreover, misretrievals of the partially cue-matching distractor (*Jonathan* in 3a) are predicted. These misretrievals are predicted to be reflected in response-accuracies if the comprehension questions target the reflexive-antecedent dependency. For conditions with a mismatching antecedent (see 3c, d), the unconstrained cue-based retrieval model (Lewis and Vasishth, 2005) also predicts cue-based retrieval interference due to a cue-matching distractor (3c). As in antecedent-match conditions, this retrieval interference is predicted to be reflected in a higher proportion of misretrievals of the matching distractor. But, in contrast to antecedent-match conditions, no inhibition between the antecedent and the distractor is predicted because they do not share any of the experimentally manipulated retrieval relevant features (in 3c and d, the antecedent and the distractor neither share the gender cue {gender:fem} nor the structural cue {c-com:+}). Since ACT-R predicts faster retrieval latencies in the case of misretrievals as a result of a race-like configuration, the trials with misretrievals are predicted to lead to a decreased mean retrieval latency. Therefore, in the absence of inhibition between the distractor and the antecedent in antecedent-mismatch conditions, faster processing times are predicted when a feature-matching distractor is present.

Encoding interference predicts increased retrieval latencies and a higher proportion of misretrievals as a function of the number of features the target (here the antecedent) shares with other items in memory (Oberauer and Kliegl, 2006).^6^ Thus, in conditions with a matching antecedent (see 3a,b), a slow-down and a higher proportion of misretrievals due to a feature-matching distractor (3a) is expected. By contrast, in conditions with a mismatching antecedent (see 3c,d), a slow-down and a higher proportion of misretrievals due to a *feature-mismatching* distractor (3d) is predicted since the mismatching antecedent and the mismatching distractor have the same gender feature {gender:masc}.^7^

To summarize, for materials as the ones presented in (3), both encoding interference and cue-based retrieval interference predict that a matching distractor leads to a processing slow-down in antecedent-match conditions and to a speed-up in antecedent-mismatch conditions. For online reading time measures, both accounts thus make precisely the same predictions and can account for the inhibitory effects in antecedent-match conditions reported by Badecker and Straub (2002), Chen et al. (2012), Clackson and Heyer (2014), Jäger et al. (2015) and Patil et al. (unpublished manuscript) as well as for the facilitatory effects in antecedent-mismatch conditions reported by King et al. (2012) and Parker and Phillips (2014). For retrieval probabilities (to be reflected in response accuracies of adequate comprehension questions), both accounts also make the same predictions for antecedent-match conditions but differ in their predictions for antecedent-mismatch conditions. Hence, if online evidence for encoding interference in reflexives can be found, we need to reconsider the theoretical implications of interference effects in reflexives with respect to the debate about structurally-guided vs. unconstrained cue-based retrieval. In the following, we present two experiments on German and one experiment on Swedish designed to disentangle encoding from cue-based retrieval interference.

## 2. Experiment 1: german reflexives (self-paced reading)

The German reflexive *sich* “himself”/“herself” is an interesting test case for teasing apart encoding from cue-based retrieval interference. The third-person singular reflexive *sich* is gender neutral and, roughly speaking, requires its antecedent to be a c-commanding noun phrase contained in the reflexive's local clause. For more details about the syntactic properties of German reflexives see Everaert (1986), Reinhart and Reuland (1993), Reuland and Reinhart (1995), Reuland (2001), Gast and Haas (2008) and Reuland (2011). Since *sich* is gender neutral and thus gender can be assumed to not be used as a retrieval cue, we do not expect any cue-based retrieval interference from a structurally inaccessible distractor that shares its gender with the antecedent. Encoding interference, in contrast, predicts that a distractor of the same gender as the antecedent leads to a degradation of the antecedent's memory representation resulting in longer processing times when retrieving the antecedent upon encountering the reflexive. Moreover, encoding interference predicts a lower retrieval probability of the antecedent when a gender-sharing distractor is present. We will use the term *gender-overlap* to refer to the situation where the antecedent and the distractor share their gender in order to reserve the term *gender-match* for the match of an item's feature with a retrieval cue as in Example (3) discussed above.

### 2.1. Materials and method

#### 2.1.1. Materials

The experimental items consist of a matrix clause whose subject is the antecedent of the third person singular reflexive *sich* (see 4 for an example). The reflexive is the first constituent of a conjoint determiner phrase (*sich und die Kollegen* in 4) which as a whole is the direct object of the matrix verb. The antecedent (*der Dieb/die Diebin* in 4) is modified by an object-extracted relative clause that intervenes between the antecedent and the reflexive. The subject of this relative clause (*der Hehler/die Hehlerin* in 4) does not c-command the reflexive and hence syntactically disqualifies as antecedent. We will refer to this noun phrase as *distractor*. Both the antecedent and the distractor were always animate common nouns with a definite article. King et al. (2012) have shown that interference effects in reflexives are more likely to be detected when the verb, which triggers the retrieval of its subject—which, in turn, is also the reflexive's antecedent— does not directly precede the reflexive. In order to increase the chances of detecting an effect, we chose perfective tense for our materials, because, as opposed to present tense or simple past, the reflexive precedes the main verb in perfective sentences (for another study on interference effects using pre-verbal reflexives see Kush and Phillips, 2014). Moreover, we inserted a relatively long adverb between the perfective auxiliary *hat* and the reflexive. As in the classical gender-match/mismatch design, we manipulated the antecedent's and the distractor's gender. This resulted in a fully crossed 2×2 design with gender of the antecedent (masculine vs. feminine) and interference (gender-overlap vs. no gender-overlap between the distractor and the antecedent) as factors. For our research question, the gender manipulation of the antecedent was not of interest *per se*. It was included in order to experimentally control for lexical properties such as word length or frequency which, due to the nature of the German language, are inseparable from the gender manipulation. We will discuss this issue more in detail in the Results section.

**Table:** 

(4)	**Masc/Fem antecedent; Masc/Fem distractor**
	***Der Dieb**_i_**/Die Diebin**_i_, dem/der **der Hehler**_j_**/die Hehlerin**_j_ befohlen hat zu stehlen, hat*
	**the thief-masc/the thief-fem** whom **the dealer-masc/the dealer-fem** obliged has to steal has
	überraschenderweise **sich**_i/*j_ und die Kollegen angezeigt, berichtete das Hochglanzmagazin.
	surprisingly **self** and the colleagues denounced reported the magazine
	‘The thief whom the dealer obliged to steal surprisingly denounced himself/herself and the colleagues, reported the magazine.’

Each sentence was followed by a *yes/no* comprehension question targeting the reflexive-antecedent dependency. One half of the comprehension questions tested whether the antecedent was retrieved successfully (to be answered with *yes*) and the other half tested whether the distractor was misretrieved instead (to be answered with *no*). Question types were balanced across items and held constant within the four conditions of each item.

#### 2.1.2. Participants and procedure

144 undergraduate students from the University of Potsdam who were all native speakers of German participated in the study for credit or payment of 5 EUR. We chose a relatively large sample size in order to increase statistical power, i.e., reduce Type II error probability. For our research question, high statistical power is particularly important since if encoding interference in the processing of reflexives is absent, a null result is predicted. The number of participants was determined based on a statistical power test assuming an effect of 20 ms and a standard deviation of 75 ms. In order to achieve power of 90%, 149 participants would be needed. Due to the restricted nature of our participant pool, the actual sample size was slightly smaller, which yielded a statistical power of 0.89%. 16 test items and 32 filler sentences were presented in a moving-window self-paced reading paradigm (Just et al., 1982). Items were arranged according to a Latin Square with a different randomization for each participant. Each trial was followed by a *yes/no* comprehension question.

### 2.2. Results

Statistical analyses were carried out in GNU-R (R Development Core Team, 2011) using linear mixed effects models provided by the lme4 package version 1.0-6 (Bates et al., 2014). Binary dependent variables were modeled using generalized linear mixed models with a logistic link function. For the analyses of comprehension questions and reading times, we fit models testing for a main effect of gender of the antecedent, a main effect of interference (i.e., effect of whether or not the distractor overlapped in gender with the antecedent) and an interaction between the two. All models were fit with random intercepts and slopes for participants and items (Baayen et al., 2008). No correlations between random effects were estimated since in many of the models the correlation matrix of random effects was degenerate.

In German, the feminine form of a noun is usually generated by adding the suffix -*in* and in many nouns, the masculine form is more frequent than the feminine one. Therefore, a correlation between gender and word length and word frequency could not be avoided in the stimuli. More precisely, correlations between the main effect of gender and frequency/length of the antecedent as well as correlations between the interaction antecedent gender × interference and frequency/length of the distractor are expected. Crucially, including the gender manipulation of the antecedent as a fully crossed within-items factor in our design ensured a zero correlation between frequency/length of the antecedent or the distractor with the critical main effect of interference. Along the same lines, correlations between frequency/length of the antecedent and the interaction antecedent gender × interference as well as correlations between frequency/length of the distractor and the main effect of gender of the antecedent cancel out due to the fully-crossed factorial design. To test these assumptions and to obtain estimates for the expected correlations, we computed Pearson-correlations of each of the contrasts to be tested in the linear-mixed model with centered word lengths measured in number of characters and centered log-transformed lemma frequencies taken from dlexDB^8^ (Heister et al., 2011) of the antecedent and the distractor (see Table 1). As expected, there was a positive correlation (*r* = 0.63) between the main effect of gender of the antecedent and frequency of the antecedent and a negative correlation (*r* = −0.44) between the main effect of gender of the antecedent and word length of the antecedent. Similarly, there was a positive correlation (*r* = 0.39) between the frequency of the distractor and the interaction antecedent gender × interference and a small negative correlation between word length of the distractor and the interaction antecedent gender × interference. Thus, a main effect of gender of the antecedent and the interaction between the two main effects should not be interpreted since they might be confounded with the effects of antecedent/distractor length and frequency.

**Table 1:** **Experiments 1 and 2**.

	**Length antecedent**	**Frequency antecedent**	**Length distractor**	**Frequency distractor**
Interference	0	0	0	0
Gender antecedent	−0.44	0.63	0	0
Gender ant. × Interf.	0	0	−0.24	0.39

Correlations (Pearson correlation coefficient) of word length and log lemma frequency of the antecedent and the distractor with the experimental manipulations (main effect of interference, main effect of gender of the antecedent and their interaction). Word length and log lemma frequencies were centered (z-scores).

#### 2.2.1. Comprehension questions

Comprehension question response accuracies were analyzed using a linear mixed model with a logistic link function. Mean accuracy scores of question responses are provided in Table 2. Statistical analyses revealed a main effect of interference: accuracy was lower in conditions with a gender-sharing distractor (estimate = −0.25, *SE* = 0.12, *z* = −2.02, *p* < 0.05). Neither the main effect of gender nor the interaction were significant.

**Table 2:** **Experiment 1**.

**Condition**	**Accuracy**
Gender-overlap - masculine antecedent	71
Gender-overlap - feminine antecedent	73
No gender-overlap - masculine antecedent	75
No gender-overlap - feminine antecedent	77

Mean accuracy scores of question responses in percentage by experimental condition.

#### 2.2.2. Reading times

An overview of raw reading times for each region of the sentence is provided in Table A1 in the Appendix. Reading times were analyzed at the reflexive, the following NP together with the preceding conjunction *und* “and” (n+1), the main clause verb (n+2) as well as at the two words preceding the reflexive as a sanity test of the baseline reading times. In order to achieve a close to normal distribution of the model residuals, we analyzed negative reciprocal reading times (Box and Cox, 1964). None of the comparisons reached significance at any region. Modeling log-transformed RTs instead of reciprocal RTs yielded similar results. The output of the linear-mixed models is provided in Table 3.

**Table 3:** **Experiment 1**.

	**n−1**			**REFL**			**n+1**			**n+2**		
**Predictor**	***coef***	***SE***	***t***	***coef***	***SE***	***t***	***coef***	***SE***	***t***	***coef***	***SE***	***t***
Interference	4e-05	3e-05	1.39	2e-05	2e-05	0.67	0	1e-05	0.18	−1e-05	3e-05	−0.58
Gender antecedent	0	2e-05	−0.08	1e-05	3e-05	0.29	0	1e-05	−0.42	−4e-05	3e-05	−1.25
Gender ant.×Interf.	−4e-05	3e-05	−1.49	−3e-05	2e-05	−1.49	−1e-05	1e-05	−0.96	−1e-05	3e-05	−0.45

Main effects of interference and gender of the antecedent and their interaction on negative reciprocal RTs as dependent variable measured at the adverb preceding the reflexive (n−1), the reflexive (REFL), the coordinate NP following the reflexive (n+1) and the main clause verb (n+2).

### 2.3. Discussion

In reading times, we did not find any effect of gender-overlap between the antecedent and the distractor. However, in comprehension questions, we observed lower response accuracies when the distractor overlapped in gender with the antecedent. This effect might be explained by misretrievals due to encoding interference during online processing which, critically, did not affect processing times. Alternatively, the lower response accuracies in the gender-overlap conditions might reflect an offline effect that arises at the moment of answering the comprehension question. Crucially for our research question, we could not find any evidence supporting the idea that encoding interference affects online processing times at the reflexive. With respect to previous studies on reflexives, we can therefore conclude that there is no indication that the interference effects observed in previous studies reflect encoding rather than cue-based retrieval interference.

## 3. Experiment 2: german reflexives (eye-tracking)

Experiment 2 is a cross-methodological replication of Experiment 1. Already Ronald Fisher, the father of frequentist statistics, emphasized the importance of replication (Fisher, 1937, page 16). Indeed, non-replicable findings are a major problem in experimental psychology and psycholinguistics (Simmons et al., 2011; Asendorpf et al., 2013). Moreover, a potential concern about Experiment 1 is that our conclusions are based on a null result. Although we have addressed this issue by testing a large sample and thus gaining high statistical power, one could still argue that the self-paced reading method is not sensitive enough to detect a potential effect. We therefore tested the same materials as in Experiment 1 in an eye-tracking while reading paradigm, which presumably is a more sensitive method compared to self-paced reading (Staub and Rayner, 2007).

### 3.1. Materials and method

#### 3.1.1. Materials

The same stimuli (including fillers) were used as in Experiment 1.

#### 3.1.2. Participants and procedure

151 undergraduate students from the University of Potsdam with normal or corrected-to-normal vision who were all native speakers of German participated in the experiment against credit or payment of 7 EUR. None of the participants had participated in Experiment 1.

Participants' eye movements (right eye monocular tracking) were recorded with an SR Research Eyelink 1000 eyetracker at a sampling rate of 1000 Hz using a desktop mount camera system with a 35 mm lens. The participant was seated at a height-adjustable table with his/her head stabilized using a forehead/chin-rest. Stimuli were presented on a 22 inch monitor (resolution of 1680 × 1050 pixels) with an eye-to-screen distance of 62 cm and an eye-to-camera distance of 60 cm. As a response pad, a Microsoft Button Box was used. Stimuli were presented using Experiment Builder software provided by SR Research. The experimental items were presented on a light gray background in black font, font type Times New Roman, font size 14. They were arranged according to a Latin Square and were pseudo-randomized for each participant separately such that every experimental trial was preceded by at least one filler sentence. A nine-point calibration was carried out at the beginning of the experiment and repeated during the experiment, if needed. Each experimental session started with 6 practice trials. At the beginning of each trial, participants had to fixate a drift correction point at the left center of the screen where the first word of the sentence was to appear.

### 3.2. Results

Linear mixed-effects models were fit with the same predictors as for Experiment 1. As in the analysis of Experiment 1, all models were fit with varying intercepts and slopes for participants and items. No correlations between random effects were estimated since, as in the data of Experiment 1, the correlation matrix of random effects was degenerate in many of the models.

#### 3.2.1. Comprehension questions

Mean accuracy scores by experimental condition are provided in Table 4. We observed a marginal main effect of interference with lower accuracies in conditions where antecedent and distractor had the same gender (estimate = −0.20, *SE* = 0.10, *z* = −1.95, *p* = 0.05). This replicates the pattern found in Experiment 1. None of the other effects was significant.

**Table 4:** **Experiment 2**.

**Condition**	**Accuracy**
Gender-overlap - masculine antecedent	74
Gender-overlap - feminine antecedent	69
No gender-overlap - masculine antecedent	75
No gender-overlap - feminine antecedent	75

Mean accuracy scores of question responses in percentage by experimental condition.

#### 3.2.2. Eye movements

An overview of raw reading times at each word of the sentence is provided in Table A2 in the Appendix. The same regions were analyzed as in Experiment 1. Raw fixation durations shorter than 20 ms or longer than 1000 ms (0.25% of the data) were excluded from all analyses. In eye-tracking data, the dependent measures can be partitioned into first-pass, regression-related (proportions of regressions and duration of regressive events) and later-pass measures. Since the exact mapping between syntactic effects and eye-tracking measures is still unclear (Clifton et al., 2007), we analyzed one representative measure from each group. As a first-pass measure, we analyzed first-pass reading time (FPRT, also referred to as gaze duration), which is defined as the sum of all first-pass fixations on a region. As regression related measures, we analyzed first-pass regression-probability (FPRP), i.e., a binary variable coded as 1 if a first-pass regression was initiated from a region, and regression-path duration (RPD), i.e., the sum of all fixation durations starting from the first fixation on a region until leaving this region to the right including all regressive fixations that fall into this time window. As a later-pass measure, we analyzed total-fixation time (TFT), i.e., the sum of all fixations on a region. Strictly speaking, TFT is not a pure late measure but rather the sum of FPRT and re-reading time. However, we chose to report TFT as a representative late measure since TFT is one of the most commonly reported measures in psycholinguistics; we do not analyze re-reading time because the critical region was re-read in only about 20% of the trials leading to very low statistical power. In order to achieve approximately normally distributed residuals, the continuous dependent variables were log-transformed (Box and Cox, 1964).

An overview of the output of the linear mixed-effects models is provided in Table 5. At the reflexive (n), the word preceding the reflexive and the region following the reflexive, none of the comparisons reached significance in any of the dependent variables. At region n+2 (i.e., the main clause verb), a significant effect of gender of the antecedent was observed in TFT (longer fixation times in conditions with a feminine antecedent). However, as we have argued in the Results section of Experiment 1, this effect should not be interpreted since it correlates with frequency and length of the antecedent. For our research question, only the main effect of interference is relevant.

**Table 5:** **Experiment 2**.

**DV**	**Predictor**	**n−1**			**REFL**			**n+1**			**n+2**		
		***coef***	***SE***	***t or z***	***coef***	***SE***	***t or z***	***coef***	***SE***	***t or z***	***coef***	***SE***	***t or z***
FPRT	Interference	−0.01	0.02	−0.25	−0.01	0.02	−0.31	−0.01	0.03	−0.38	−0.02	0.01	−1.14
	Gender antecedent	0.01	0.02	0.61	−0.03	0.02	−1.35	−0.03	0.03	−1.03	0.01	0.01	0.64
	Gender ant.×Interf.	−0.02	0.02	−1.06	−0.01	0.02	−0.64	−0.04	0.02	−1.91	0	0.02	0.25
RPD	Interference	0.04	0.03	1.59	0.01	0.02	0.44	−0.02	0.03	−0.67	0	0.03	−0.06
	Gender antecedent	0.02	0.02	0.86	−0.02	0.02	−0.76	−0.02	0.02	−1.01	0.01	0.02	0.55
	Gender ant.×Interf.	−0.03	0.02	−1.14	0.03	0.02	1.1	−0.02	0.02	−1.01	0	0.02	−0.22
FPRP	Interference	0.19	0.11	1.64	0.12	0.14	0.83	−0.05	0.11	−0.44	0.13	0.17	0.77
	Gender antecedent	0.09	0.12	0.72	−0.1	0.14	−0.7	−0.05	0.14	−0.34	−0.01	0.13	−0.05
	Gender ant.×Interf.	0.03	0.11	0.22	0.18	0.18	0.98	0.1	0.11	0.85	−0.01	0.14	−0.09
TFT	Interference	0.03	0.02	1.4	−0.01	0.02	−0.36	0.01	0.02	0.37	0	0.02	0.12
	Gender antecedent	0.03	0.02	1.54	0.02	0.02	0.68	0	0.02	0.13	0.04	0.02	2.22*
	Gender ant.×Interf.	0.01	0.02	0.29	0.01	0.02	0.46	−0.04	0.02	−1.93	−0.01	0.02	−0.72

Main effects of interference and gender of the antecedent and their interaction on the dependent variables log-first-pass reading time, log-regression-path duration, first-pass regression probability and log-total fixation time measured at the adverb preceding the reflexive (n−1), the reflexive (REFL), the coordinate NP following the reflexive (n+1) and the main clause verb (n+2). Statistically significant (α = 0.05) effects are marked with an asterisk.

Moreover, a *post-hoc* analysis of the region containing the relative clause verb (*zu stehlen* in Example 4) revealed a significant main effect of interference in TFT with longer fixation durations when the antecedent and the distractor overlapped in gender (estimate = 0.05, *SE* = 0.02, *t* = 2.28).

### 3.3. Discussion

Experiment 2 replicated the findings of Experiment 1. As in Experiment 1, no evidence for encoding interference due to gender-overlap between the reflexive's antecedent and a structurally inaccessible distractor was observed neither at the reflexive, nor at the pre- or post-critical regions.

At the relative clause verb, however, gender-overlap between the main clause subject, i.e., the antecedent, and the relative clause subject, i.e., the distractor, led to significantly longer total-fixation times. At this region, the relative clause subject needs to be retrieved. Hence, the observed effect, which appears in a similar region as the effects reported by Gordon et al. (2001), might reflect encoding interference. However, it is disconcerting that this effect was observed only in total-fixation time and was not present in Experiment 1, as a *post-hoc* analysis of the self-paced reading data showed. Thus, one might discount this effect as a possible Type I error. If one does not discount the effect, it raises the question why encoding interference affects argument-head dependency completion, but not reflexive-antecedent dependency formation. A possible explanation might be that the encoding interference effect (to the extent that it is not a Type I error) dies out by the time the reflexive is processed.^9^ In any case, further replication attempts of this configuration are needed. In sum, it is possible that we are seeing encoding interference at the distractor, but, which is crucial for our research question, this encoding interference does not seem to have any effect at the reflexive.

Taken together, the results of Experiments 1 and 2 are a strong indication that in reflexive-antecedent dependency formation, the sharing of a non-structural feature such as gender does not lead to encoding interference reflected in a processing slow-down. More precisely, it indicates that in materials of the type used in this experiment, encoding interference does not affect retrieval latencies of the antecedent when processing the reflexive. However, the marginal interference effect in offline comprehension accuracies, which had been significant in Experiment 1, indicates that the antecedent was retrieved less often correctly when it shared its gender with the distractor. This can be interpreted as evidence for encoding interference affecting retrieval probability of the antecedent. In sum, neither experiment provides any evidence for the claim that encoding interference affects reading time at the reflexive. However, our offline results suggest that encoding interference might affect retrieval probability of the antecedent. Crucially, even if encoding interference affected retrieval probability of the antecedent or the offline interpretation of the sentence, there is no evidence that it affects the participants' online behavior at the reflexive measured in self-paced reading times or eye-movements. Hence, encoding interference is not a plausible explanation for the *online* effects previous studies have observed in eye-tracking or self-paced reading measures.

## 4. Experiment 3: swedish possessives (eye-tracking)

Experiments 1 and 2 yielded converging results: we found no evidence for encoding interference affecting the online processing speed of German reflexives. However, there are still two potential concerns with these results: (i) Our conclusion is based on two null-results, and (ii) we need to cross-linguistically validate our conclusion. In Experiment 3, we addressed these issues by examining the processing of Swedish possessives in an eye-tracking experiment. In Swedish, there are two kinds of possessives: reflexive possessives that are not gender-marked and pronominal possessives that need to agree in gender with their antecedent. The reflexive possessive *sin* “his”/“her” can only be bound by a c-commanding antecedent inside its local clause. In contrast, the pronominal possessive *hans* “his” must not be bound within its local clause, but requires an antecedent outside its clause domain (see Holmes and Hinchliffe, 1994 and Kaiser, 2003, p. 209). In a 2 × 2 factorial design, we manipulated anaphor type (pronominal possessive vs. reflexive possessive) and interference, i.e., whether or not a structurally inaccessible distractor shared the gender of the antecedent. For this design, encoding interference predicts increased processing difficulty in the gender-overlap conditions compared to the no-gender-overlap conditions, regardless of anaphor type. Cue-based retrieval interference, in contrast, predicts an interaction between anaphor type and interference: increased processing difficulty due to a gender-sharing distractor is predicted for the gender-marked pronominal possessives but not for the gender-unmarked reflexive possessives. This is because only in pronominal possessives, the gender-marked anaphor can trigger a retrieval process where gender is used as a retrieval cue. When both the antecedent and the distractor match the gender cue, cue-based retrieval interference predicts inhibition between the antecedent and distractor and a higher proportion of misretrievals of the distractor (Lewis and Vasishth, 2005). Thus, the present experiment allows us to directly pit encoding and cue-based retrieval interference against each other. In contrast to Experiments 1 and 2, cue-based retrieval interference predicts an interaction rather than a null-result.

**Table:** 

(5)	a. **Pronominal possessives; gender-overlap/no gender-overlap**
	**Åke**_i_ säger att **Alf**_j_**/Ann**_j_ jobbade med **hans**_i/*j_ sysslingar på helgerna.
	**Åke-masc** says that **Alf-masc/Ann-fem** worked with **his** cousins at the weekend
	‘Åke says that Alf/Ann worked with his cousins at the weekend.’
	b. **Reflexive possessives; gender-overlap/no gender-overlap**
	**Åke**_i_ som **Alf**_j_**/Ann**_j_ tackade ringer **sina**_i/*j_ sysslingar på kvällen.
	**Åke-masc** whom **Alf-masc/Ann-fem** thanked calls **his** cousins in the evening
	‘Åke, whom Alf/Ann thanked, calls his cousins in the evening.’

### 4.1. Materials and method

#### 4.1.1. Materials

The conditions with pronominal possessives (see 5a for an example item) consist of a superordinate clause whose subject is the antecedent (*Åke* in 5a) and a subordinate clause containing the distractor (*Alf* or *Ann* in 5a) which either matches or mismatches the gender of the antecedent and the gender-marked pronominal possessive (*hans* “his” in 5a). The conditions with reflexive possessives (see 5b for an example item) consist of a main clause containing the antecedent (*Åke* in 5b) and the gender-unmarked reflexive possessive (*sina* “his”/“her” in 5b). The distractor (*Alf* or *Ann* in 5b) is the subject of an appositive relative clause intervening between the antecedent and the reflexive possessive. As Swedish does not code masculine and feminine as grammatical gender, and the number of nouns with inherent gender such as *boy* or *girl* is very limited, both the antecedent and the distractor were proper names in all experimental sentences. Indeed, it is crucial for our research question to extend the findings of Experiments 1 and 2 to proper names, which differ from common nouns with respect to their referential properties (Longobardi, 1994; Elbourne, 2005), since several of the studies reporting interference effects in reflexives actually employed proper names (e.g., Badecker and Straub, 2002).

The nouns used as antecedents and distractors are all highly frequent, gender unambiguous Swedish first names taken from Statistics Sweden, a database which contains the 100 most frequently given and used male and female first names in Sweden.^10^ Antecedents and distractors are all matched for word length (numbers of characters) within each item. Half of the items have a feminine antecedent and the other half a masculine antecedent. The possessed noun phrase (*sysslingar* in 5) is always a plural noun.

Two types of comprehension questions were designed. The first type probed for the correct interpretation of the anaphor-antecedent dependency. 50% of these questions were to be answered with *yes*. The second question type targeted various parts of the sentence, but not the interpretation of the anaphor. Again, 50% of these questions were to be answered with *yes*.

#### 4.1.2. Participants and procedure

35 native speakers of Swedish currently living in Berlin or Potsdam with normal or corrected-to-normal vision participated in the experiment against payment of 5 EUR (plus 6.20 EUR to cover travel expenses). The sample size was smaller compared to Experiments 1 and 2 due to logistic limitations, but we tested a larger number of experimental items compared to Experiments 1 and 2. Participants' eye movements (right eye monocular tracking) were recorded while reading 48 experimental sentences and 70 filler sentences. The general technical set-up was the same as in Experiment 2. Stimuli were arranged in a Latin Square and pseudo-randomized such that each experimental trial was preceded by at least one filler sentence. Each trial was followed by a comprehension question. Two thirds of the comprehension questions targeted the correct interpretation of the anaphor and one third targeted other parts of the sentence. The experiment started with 5 practice trials to familiarize participants with the procedure.

### 4.2. Results

On all dependent variables, we fit linear mixed-effects models with main effects of anaphor type (pronominal vs. reflexive possessive), interference (whether or not the distractor had the same gender as the antecedent) and their interaction as predictors. When the interaction reached significance, nested contrasts testing for an interference effect within each anaphor type were fit. All models were fit with varying intercepts for participants and items. No varying slopes were fit because the generalized likelihood-ratio test showed that they did not improve the model fit. The pattern of results was not affected by whether or not varying slopes were fit. For the interpretation of results, it should be kept in mind that the effect of anaphor type is not of theoretical relevance to our research question. As the two levels of anaphor type differ lexically at the pre-critical and the critical region, a main effect of anaphor type does not have any useful interpretation.

#### 4.2.1. Comprehension questions

Mean accuracy scores by experimental condition and question type (i.e., whether or not the comprehension question targeted the anaphor) are provided in Table 6. We ran a linear-mixed effects model with a logistic link function with main effects of anaphor type, interference and question type and their interactions including the three-way interaction between all main effects as predictors. The model output is summarized in Table 7. The main effect of interference and the interaction between interference and question type reached significance. Moreover, a marginal three-way interaction between interference, anaphor type and question type was observed. A second model in which we applied nested contrasts testing for an interference effect within each level of anaphor type and question type^11^ showed that the interactions were caused by a highly significant interference effect that was present only in questions targeting the anaphor in pronominal possessives (estimate = −1.16, *SE* = 0.25, *z* = −4.62, *p* < 0.0001). In sum, in questions targeting the anaphor-antecedent dependency, the presence of a gender matching distractor led to lower response accuracies in sentences with pronominal possessives but not in sentences with reflexive possessives. In questions not targeting the anaphor-antecedent dependency, no effects were observed.

**Table 6:** **Experiment 3**.

**Condition**	**Accuracy**	
	**Anaphor**	**Other**
Pronominal - gender-overlap	75	82
Pronominal - no gender-overlap	90	82
Reflexive - gender-overlap	85	80
Reflexive - no gender-overlap	86	81

Mean accuracy scores of comprehension questions in percentage by experimental condition and question type, i.e., whether the question targeted the anaphor-antecedent dependency or another element of the sentence.

**Table 7:** **Experiment 3**.

**Predictor**	***coef***	***SE***	***z***
Interference	−0.17	0.07	−2.42*
Anaphor type	−0.01	0.07	−0.17
Question type	0.12	0.10	1.11
Interference×Anaphor type	−0.12	0.07	−1.61
Interference×Question type	−0.15	0.07	−2.04*
Anaphor type×Question type	−0.06	0.07	−0.86
Interference×Anaphor type×Question type	−0.14	0.07	−1.95

Analysis of comprehension questions: Main effects of interference, anaphor type and question type together with their interactions. Statistically significant (α = 0.05) effects are marked with an asterisk.

#### 4.2.2. Eye movements

An overview of raw reading times at each region of the sentence is provided in Table A3 in the Appendix. We analyzed the pre-critical region containing the verb (plus postposition), the critical region containing the pronominal or reflexive possessive and the post-critical region containing the possessed noun. The same dependent variables were analyzed as in Experiment 2. Continuous dependent variables were log-transformed in order to achieve approximately normally distributed residuals.

An overview of the output of the linear mixed-effects models is provided in Table 8. The effect of anaphor type reached significance across regions and dependent variables. However, as mentioned above, this effect was not of interest to our research question: conditions with pronominal and reflexive possessive differ from each other in syntactic structure, distractor position, lexicon, word length and number of words contained in the pre-critical region. At the pre-critical and the critical region, no other effect reached significance in any dependent variable. At the post-critical region, a significant interaction between anaphor type and interference was observed in FPRP. Pairwise comparisons revealed that this interaction was driven by a significant interference effect in pronominal possessives. When the distractor shared the gender of the antecedent and hence matched the gender-cue, less first-pass regressions were observed (estimate = −0.44, *SE* = 0.18, *z* = −2.47, *p* < 0.05). In order to test whether this facilitation due to a gender-matching distractor reflected misretrievals of the latter, we re-ran the models on comprehension question response accuracies for trials with and without a first-pass regression from the post-critical region separately.

**Table 8:** **Experiment 3**.

**DV**	**Predictor**	**n−1**			**REFL/PRON**			**n+1**		
		***coef***	***SE***	***t or z***	***coef***	***SE***	***t or z***	***coef***	***SE***	***t or z***
FPRT	Interference	0.02	0.02	0.75	0.01	0.02	0.57	−0.03	0.02	−1.29
	Anaphor type	0.03	0.02	1.43	−0.04	0.02	−1.97	0.05	0.02	1.99
	Anaph. type×Interf.	0.03	0.02	1.28	−0.01	0.02	−0.41	0.02	0.02	0.71
RPD	Interference	−0.01	0.03	−0.18	−0.02	0.03	−0.67	0	0.04	−0.05
	Anaphor type	−0.25	0.03	−8.11*	−0.13	0.03	−4.04*	−0.07	0.04	−1.94
	Anaph. type×Interf.	0.01	0.03	0.29	0.01	0.03	0.17	−0.02	0.04	−0.53
FPRP	Interference	−0.08	0.13	−0.62	−0.11	0.13	−0.85	−0.19	0.12	−1.56
	Anaphor type	−1.26	0.13	−9.4*	−0.32	0.13	−2.45*	−0.4	0.12	−3.36*
	Anaph. type×Interf.	−0.08	0.13	−0.63	0.09	0.13	0.7	−0.25	0.12	−2.1*
TFT	Interference	0.04	0.03	1.46	0.02	0.03	0.64	0.01	0.03	0.5
	Anaphor type	−0.07	0.03	−2.44*	0.09	0.03	3.15*	0.03	0.03	0.89
	Anaph. type×Interf.	0.05	0.03	1.7	−0.01	0.03	−0.4	0.02	0.03	0.65

Main effects of interference, anaphor type and their interaction at the pre-critcal region n−1, the reflexive/pronominal possessive (REFL/PRON) and the post-critical region n+1. The dependent measures are log-first-pass reading time, log-regression-path duration, first-pass regression probability and log-total fixation time. Statistically significant (α = 0.05) effects are marked with an asterisk.

In trials without a first-pass regression from n+1, the interference effect in pronominal possessives in questions targeting the critical dependency (i.e., the effect observed in the overall data) was highly significant (estimate = −1.19, *SE* = 0.28, *z* = −4.21, *p* < 0.0001). By contrast, in trials with a first-pass regression initiated at n+1, this effect did not reach significance (estimate = −0.94, *SE* = 0.57, *z* = −1.66, *p* = 0.09). This *post-hoc* analysis clearly shows that the interference effect in response accuracies in pronominal possessives was driven by trials in which no first-pass regression was initiated, i.e., by the trials responsible for the facilitation observed in FPRP.

### 4.3. Discussion

We did not find any evidence for encoding interference affecting processing times of Swedish anaphor-antecedent dependencies. Together with the results of Experiments 1 and 2, this suggests that in materials with a classical gender-match/mismatch manipulation, encoding interference does not affect retrieval latencies of the antecedent. In comprehension questions, we did not see evidence for encoding interference affecting retrieval probability of the antecedent either. This is in contrast to the pattern observed in response accuracies of Experiments 1 and 2.

Evidence for interference occurring at the moment of retrieval was observed in online and offline measures. The lower proportion of first-pass regressions initiated at the region directly after the gender-marked pronominal possessive in conditions with a gender-matching distractor indicates a processing facilitation due to a cue-matching distractor. Such a facilitation can be explained in terms of misretrievals of the gender-matching distractor under the assumption that misretrievals go along with shorter retrieval latencies. The lower response accuracies in comprehension questions targeting the retrieval of the antecedent support this explanation. Indeed, the *post-hoc* analysis of response accuracies for trials with and without a first-pass regression from the post-critical region clearly shows that the facilitation observed in first-pass regressions is directly connected to misretrievals of the gender-matching distractor.

The cue-based ACT-R model of sentence processing (Lewis and Vasishth, 2005) predicts misretrievals of the gender-matching distractor. These misretrievals are predicted to lead to shorter retrieval latencies, i.e., a processing facilitation, in the respective trials. However, the ACT-R model also predicts inhibition between the gender-matching distractor and the antecedent leading to longer retrieval latencies of the antecedent. Overall, the predicted direction of the interference effect therefore depends on the concrete parameter setting of the model. With the default parameter setting, inhibitory interference (i.e., the opposite effect than the one in the data) is predicted. If one assumes a particularly high activation of the distractor, ACT-R predicts the observed pattern because the highly activated distractor is misretrieved in a considerable proportion of the trials, which leads to a speed-up in the observed mean retrieval latencies (Jäger et al., 2015). Indeed, facilitation in a configuration similar to our materials has been observed in previous studies (Sturt, 2003; Cunnings and Felser, 2013; Laurinavichyute et al., 2015; Patil et al., unpublished manuscript). An argument favoring the assumption that the distractor is highly activated in our materials is that, similar to the other experiments reporting facilitation, the distractor is in subject position. Moreover, the distractor has a recency advantage over the antecedent as it is linearly closer to the retrieval site. Indeed, ACT-R predicts a recency advantage which follows from the assumption that an item's activation level decreases as a function of the passage of time (decay) and intervening material (interference). In sum, under the plausible assumption that the distractor is highly activated in our materials, cue-based retrieval interference as implemented in the ACT-R model can account for the observed pattern. Hence, the interference effect in pronominal possessives can be interpreted as evidence favoring a cue-based retrieval mechanism. However, it should be kept in mind that pronominal possessives are not subject to Binding Principle A (Chomsky, 1981). Hence, the observed effects cannot be interpreted as evidence against theories of sentence processing claiming that Principle A is immune to interference from structurally illicit antecedents (Nicol and Swinney, 1989; Phillips et al., 2011; Dillon et al., 2013).

An alternative explanation that can account for the facilitation leading to misretrievals of the gender-matching distractor in pronominal possessives but not in reflexive possessives builds on the fact that we are comparing reflexive possessives which are subject to Binding Principle A with pronominal possessives which are subject to Binding Principle B. As mentioned above, pronominal possessives must not be bound in their local domain (Binding Principle B, see Chomsky, 1981). In the syntax-semantic literature about the interpretation of pronouns, it has been proposed that in the presence of a local c-commanding noun phrase which matches the gender feature of the anaphor (as the gender-matching distractor in the pronominal possessives conditions of Experiment 3), local binding is preferred over a non-local antecedent (Fox, 1998; Heim and Kratzer, 1998). This leads to a temporary violation of Binding Principle B. Only after the local binder has successfully been inhibited, the actual search for the structurally licit antecedent is initiated (Grodzinsky and Reinhart, 1993; Reinhart, 2000; Reuland, 2011). If in our materials, the syntactically local binder of the pronominal possessive (i.e., the distractor) is accessed in a first stage of the retrieval process, in a certain proportion of the trials, this local binder might be misretrieved in case it matches the gender of the pronominal possessive and the search for the antecedent terminates already after this first stage. Such a scenario would explain the misretrievals reflected in response accuracies and also the speed-up in trials where misretrievals occurred. This model correctly predicts that facilitatory interference should be observed only with Principle B pronouns, not with Principle A reflexives since in reflexives, the local binder is the licit antecedent. Crucially, the absence of an effect in our reflexive possessive conditions is not explained by them being unmarked for gender but rather by their syntactic binding properties.

To summarize, we found no evidence for encoding interference affecting the processing of Swedish possessives. We did observe evidence for retrieval interference in gender-marked pronominal possessives. The presence of a gender-matching distractor led to facilitated processing, presumably as a consequence of misretrievals of the latter in a certain proportion of trials. Although this pattern can be explained in terms of unconstrained cue-based retrieval, it is also consistent with the view that comprehending a pronoun constrained by Principle B requires comprehenders to temporarily consider and inhibit coreference with the local subject (the distractor in our materials). However, it should be noted that recent evidence from English pronouns reported by Chow et al. (2014) is inconsistent with the idea of first accessing and subsequently inhibiting a local antecedent. In none of their five reading experiments did they observe a facilitatory effect on pronoun resolution from a feature-matching local antecedent.

## 5. General discussion

We set out to find evidence for encoding interference in the processing of reflexives. With respect to the current debate about structure-based vs. unconstrained cue-based retrieval subserving the processing of reflexives, the question whether encoding interference can be observed in reflexives is crucial because, as has been argued by Dillon (2011), encoding interference provides an alternative explanation for interference effects in reflexives which originally have been attributed to cue-based retrieval interference and hence taken as evidence for unconstrained cue-based retrieval (Badecker and Straub, 2002; Chen et al., 2012; Jäger et al., 2015; Patil et al., unpublished manuscript).

In order to decide whether encoding interference is present in the processing of reflexives, we conducted two experiments on the German reflexive *sich*. In contrast to previous studies, where encoding and cue-based retrieval interference made the same predictions, the gender-unmarked *sich* allowed us to pit against each other the predictions of retrieval and encoding interference. Cue-based retrieval interference predicts no effect of gender of a structurally inaccessible distractor whereas encoding interference predicts a slow-down when the gender of the distractor matches the gender of the antecedent. Neither with self-paced reading nor with eye-tracking did we find any indication for an online interference effect caused by a gender-sharing distractor, although the statistical power of our experiments was considerably higher than the one of previous experiments reporting interference effects in reflexives. We conducted a third experiment on Swedish possessives to cross-linguistically validate our finding. The interaction between interference and anaphor type provided further support for the conclusion that sharing the gender feature with a distractor does not lead to encoding interference in the processing of reflexives. Although we did not find any evidence that encoding interference affected online processing ease, response accuracies in the comprehension questions of Experiment 1 indicate that encoding interference might have caused misretrievals of the gender-sharing distractor. However, this effect was only marginal in Experiment 2 and could not be replicated in Experiment 3. Critically, these supposed misretrievals observed in Experiment 1 are not reflected in online processing measures. In sum, there is no evidence for encoding interference affecting online processing measures. Therefore, there is no evidence for the proposal that online interference effects reported in previous studies on reflexives arise from encoding interference. This finding therefore provides support for the assumption that interference effects observed in reflexive processing arise at the moment of retrieval rather than at the encoding stage. In other words, encoding interference is not a plausible explanation for reconciling interference effects with a structure-based account of reflexive processing. Thus, taken together with the interference effects reported in previous studies on reflexive processing, our findings favor an unconstrained cue-based retrieval architecture.

Lastly, we want to emphasize that our results should not be interpreted as evidence for the absence of encoding interference in sentence processing *per se*. Indeed, the effect at the relative clause verb in Experiment 2 might reflect encoding interference. The presence of encoding interference *as such* is in principle not incompatible with a content-addressable architecture since content-addressability is an architectural mechanism concerning the *retrieval*, but not the *encoding* or the *maintenance* of an item in working memory.

More generally, our findings provide support for a content-addressable memory architecture subserving language comprehension. This adds to a growing body of evidence from various kinds of syntactic dependencies such as filler-gap (McElree et al., 2003) and subject-verb dependencies (Van Dyke and Lewis, 2003; Van Dyke and McElree, 2006, 2011; Van Dyke, 2007; Wagers et al., 2009; Dillon et al., 2013), the licensing of negative-polarity items (Vasishth et al., 2008) and verb-phrase ellipsis (Martin and McElree, 2008), suggesting that the parser uses a cue-based retrieval mechanism to process these dependencies. One fundamental question in sentence processing research is whether the human parser uses qualitatively different retrieval mechanisms in the processing of different kinds of dependencies. Indeed, proponents of the structure-based account of reflexive processing have argued that the retrieval mechanisms mediating the processing of reflexives differ qualitatively from the ones used, e.g., in the processing of subject-verb dependencies (Phillips et al., 2011; Dillon et al., 2013). Hence, evidence for cue-based retrieval subserving the processing of reflexives is one important piece of evidence toward a content-addressable model of working memory underlying sentence processing in general, which not only invokes qualitatively similar working memory mechanisms to explain the processing of different kinds of linguistic dependencies, but, even beyond that, locates the language processing system within a general cognitive architecture where independently motivated working memory mechanisms operate on linguistic representations.

### Conflict of interest statement

The authors declare that the research was conducted in the absence of any commercial or financial relationships that could be construed as a potential conflict of interest.

## Footnotes

^1^Nairne (1990) did not use the term *encoding interference* but rather *feature-overwriting* to refer to his conception of interference.

^2^Note that the model proposed by McElree (2006) predicts that cue-based retrieval interference is reflected only in retrieval probability, not in retrieval latency. In contrast, the ACT-R architecture developed by Anderson and Lebiere (1998) and Anderson et al. (2004), on which the Lewis and Vasishth (2005) model of sentence processing is based, predicts retrieval interference to be reflected in both, retrieval probability and retrieval latency.

^3^The Lewis and Vasishth (2005) model *per se* does not make any commitments with respect to the question which features are used as retrieval cues. Hence it is also possible to implement the structure-based account in this framework by restricting the set of retrieval cues to structural features.

^4^It should be noted that using {c-com:+} as a feature is a simplification since a tree-configurational relation is not as straightforward to code as a feature of an item as, e.g., gender or number. For a discussion of how tree-configurational information such as c-command could be encoded as an item's feature see Alcocer and Phillips (unpublished manuscript). On a theoretical basis, Kush (2013) argues against the representation of c-command as a feature and discusses how, in online sentence processing, the human parser might distinguish between c-commanding and non-c-commanding antecedents.

^5^In some experiments, only the stereotypical gender of the accessible antecedent was violated (as in 3c,d), whereas in other studies, real feature violations were used resulting in ungrammatical sentences in the antecedent-mismatch conditions.

^6^To be precise, the number of distractors sharing a certain feature with the target also affects retrieval latencies and retrieval probability because the more distractors share this feature with the target, the higher the probability that one of these distractors “robs” this feature from the memory representation of the target.

^7^Because we set out to determine whether invoking encoding interference is a way to reconcile interference effects with structure-based retrieval, for the predictions of encoding interference we are assuming that only structural retrieval cues are used. If, by contrast, one assumes that gender is used as a retrieval cue, the feature matching distractor (3c) is predicted to be misretrieved more often than the feature mismatching distractor (3d). This prediction is orthogonal to the question of encoding interference, but follows from the basic assumption that an item's retrieval probability depends on its features' match with the retrieval cues. This basic assumption is shared by models of encoding interference (Nairne, 1990; Oberauer and Kliegl, 2006). (Note that this point is unrelated to the cue-based retrieval interference component in the Oberauer and Kliegl, 2006 model which is assumed to cause inhibition between items sharing the same retrieval cues.)

^8^www.dlexdb.de

^9^A reviewer noticed that the effect at the relative clause verb occurs in total-fixation time, a measure which can reflect processing difficulty encountered further downstream in the sentence, and therefore might actually reflect processing difficulties at the reflexive which triggers re-readings of the previous materials. However, if this were the case, one would expect an increase in the proportion of regressions or increased regression-path durations at the reflexive. As this is not the case, it is difficult to conclude that the effect observed at the verb reflects processing difficulty associated with the reflexive.

^10^http://www.scb.se/BE0001-EN; we used the data of 2012.

^11^The model predictors were main effects of anaphor and question type, interaction between anaphor type and question type and the four pairwise comparisons (interference effects in pronominal and reflexive possessives in question targeting the anaphor and questions not targeting the anaphor.)

## Appendix

**Table A1:** **Experiment 1**.

	***Der/Die***	***Dieb/-in***	***dem/der***	***der/die***	***Hehler/-in***	***befohlen***	***hat***	***zu stehlen***	***hat***	***überraschenderweise***	***sich***	***und die Kollegen***	***angezeigt***
**FPRT**													
Gend.-overlap - masc. ant.	448 (8)	547 (12)	460 (11)	412 (6)	505 (19)	523 (15)	499 (14)	669 (40)	525 (15)	482 (8)	444 (10)	1221 (16)	506 (13)
Gend.-overlap - fem. ant.	462 (12)	622 (17)	493 (10)	439 (8)	552 (16)	599 (28)	528 (16)	611 (24)	552 (19)	503 (13)	438 (6)	1233 (13)	530 (15)
No gend.-overlap - masc. ant.	482 (15)	562 (22)	459 (8)	426 (10)	535 (14)	571 (17)	515 (17)	614 (23)	517 (15)	485 (9)	446 (8)	1224 (11)	535 (12)
No gend.-overlap - fem. ant.	453 (10)	611 (15)	485 (9)	443 (9)	534 (13)	538 (12)	523 (19)	594 (22)	517 (16)	479 (9)	430 (6)	1218 (12)	539 (16)

Means and standard errors of raw reading times in ms for each region by experimental condition. Between-participants variance has been removed using (Cousineau, 2005)'s normalization with (Morey, 2008)'s correction factor.

**Table A2:** **Experiment 2**.

	***Der/Die***	***Dieb/-in***	***dem/der***	***der/die***	***Hehler/-in***	***befohlen***	***hat***	***zu stehlen***	***hat***	***überraschenderweise***	***sich***	***und die Kollegen***	***angezeigt***
**FPRT**													
Gend.-overlap - masc. ant.	257 (5)	351 (9)	288 (6)	275 (7)	333 (10)	344 (7)	272 (6)	431 (11)	301 (7)	350 (10)	286 (6)	246 (5)	348 (8)
Gend.-overlap - fem. ant.	244 (6)	481 (15)	338 (10)	293 (8)	372 (12)	347 (7)	273 (6)	424 (11)	295 (7)	357 (10)	300 (6)	251 (5)	341 (6)
No gend.-overlap - masc. ant.	250 (5)	364 (13)	296 (6)	257 (8)	402 (11)	341 (7)	270 (6)	417 (10)	295 (8)	360 (9)	292 (5)	252 (6)	356 (7)
No gend.-overlap - fem. ant.	243 (5)	468 (15)	342 (10)	308 (9)	314 (10)	344 (7)	283 (7)	421 (11)	305 (7)	351 (9)	299 (6)	252 (5)	347 (6)
**RPD**													
Gend.-overlap - masc. ant.	NA	408 (11)	336 (10)	379 (13)	434 (16)	389 (12)	384 (29)	748 (33)	373 (25)	487 (21)	375 (18)	348 (19)	449 (17)
Gend.-overlap - fem. ant.	NA	581 (17)	408 (12)	448 (16)	639 (22)	472 (20)	396 (27)	830 (43)	433 (32)	530 (27)	362 (14)	327 (12)	451 (19)
No gend.-overlap - masc. ant.	NA	415 (13)	337 (9)	326 (12)	501 (15)	413 (13)	337 (15)	677 (28)	387 (28)	471 (16)	350 (15)	330 (11)	463 (18)
No gend.-overlap - fem. ant.	NA	563 (16)	421 (16)	478 (16)	578 (22)	472 (19)	383 (24)	753 (38)	413 (37)	450 (17)	375 (15)	358 (19)	438 (15)
**FPRP**													
Gend.-overlap - masc. ant.	NA	15 (1)	7 (1)	22 (2)	17 (2)	6 (1)	12 (1)	28 (2)	8 (1)	20 (2)	11 (1)	15 (1)	14 (1)
Gend.-overlap - fem. ant.	NA	19 (2)	13 (1)	26 (2)	29 (2)	12 (1)	11 (1)	28 (2)	10 (1)	18 (2)	10 (1)	15 (1)	14 (1)
No gend.-overlap - masc. ant.	NA	17 (2)	8 (1)	16 (1)	15 (1)	10 (1)	10 (1)	26 (2)	7 (1)	17 (2)	9 (1)	14 (1)	13 (1)
No gend.-overlap - fem. ant.	NA	21 (2)	13 (1)	30 (2)	32 (2)	13 (1)	10 (1)	29 (2)	9 (1)	16 (2)	11 (1)	16 (1)	13 (1)
**TFT**													
Gend.-overlap - masc. ant.	346 (10)	542 (16)	464 (14)	406 (16)	514 (19)	549 (16)	352 (11)	663 (18)	355 (12)	554 (16)	365 (11)	309 (8)	462 (11)
Gend.-overlap - fem. ant.	370 (11)	838 (25)	603 (21)	495 (19)	713 (23)	580 (15)	351 (12)	662 (18)	342 (12)	543 (17)	351 (9)	308 (9)	444 (11)
No gend.-overlap - masc. ant.	336 (9)	535 (18)	473 (14)	324 (14)	618 (19)	530 (14)	346 (11)	617 (16)	353 (13)	528 (15)	358 (9)	306 (9)	465 (11)
No gend.-overlap - fem. ant.	366 (10)	772 (23)	594 (20)	515 (19)	582 (22)	551 (16)	353 (11)	613 (17)	348 (10)	530 (17)	362 (9)	310 (8)	436 (10)

Means and standard errors of raw first-pass reading time (FPRT), regression-path duration (RPD) and total-fixation time (TFT) in ms and first-pass regression probability (FPRP) in percentages for each region by experimental condition. From continuous dependent variables, between-participants variance has been removed using (Cousineau, 2005)'s normalization with (Morey, 2008)'s correction factor.

**Table A3:** **Experiment 3**.

	***Åke Åke***	***säger som***	***att Alf/Ann***	***Alf/Ann tackade***	***jobbade med ringer***	***hans sina***	***sysslingar sysslingar***	***på helgerna på kvällen***
**FPRT**								
Pron - gend.-overlap	381 (10)	403 (14)	257 (9)	292 (10)	421 (13)	287 (6)	327 (10)	650 (21)
Pron - no gend.-overlap	393 (11)	388 (12)	277 (11)	288 (10)	393 (11)	285 (6)	334 (11)	673 (22)
Refl - gend.-overlap	405 (11)	286 (10)	295 (10)	503 (17)	391 (12)	310 (11)	308 (11)	673 (21)
Refl - no gend.-overlap	397 (12)	293 (10)	300 (9)	512 (16)	397 (13)	297 (8)	327 (12)	659 (24)
**RPD**								
Pron - gend.-overlap	NA	553 (21)	421 (26)	432 (26)	505 (21)	400 (22)	549 (42)	3636 (133)
Pron - no gend.-overlap	NA	513 (18)	405 (34)	413 (24)	521 (26)	421 (26)	525 (32)	3370 (115)
Refl - gend.-overlap	NA	443 (20)	435 (22)	669 (31)	749 (42)	500 (37)	656 (56)	4120 (163)
Refl - no gend.-overlap	NA	443 (19)	455 (25)	659 (28)	738 (40)	568 (49)	643 (55)	3882 (149)
**FPRP**								
Pron - gend.-overlap	NA	19 (2)	20 (2)	16 (2)	11 (2)	16 (2)	17 (2)	80 (2)
Pron - no gend.-overlap	NA	18 (2)	16 (2)	15 (2)	13 (2)	16 (2)	24 (2)	81 (2)
Refl - gend.-overlap	NA	27 (2)	19 (2)	15 (2)	31 (2)	19 (2)	28 (2)	81 (2)
Refl - no gend.-overlap	NA	25 (2)	21 (2)	14 (2)	31 (2)	22 (2)	26 (2)	79 (2)
**TFT**								
Pron - gend.-overlap	856 (31)	1121 (40)	444 (23)	713 (29)	1010 (36)	601 (21)	685 (24)	1067 (30)
Pron - no gend.-overlap	799 (33)	1047 (33)	468 (25)	634 (25)	917 (30)	579 (19)	675 (24)	1128 (34)
Refl - gend.-overlap	1104 (46)	781 (32)	930 (38)	1412 (45)	1039 (34)	544 (20)	660 (24)	1133 (35)
Refl - no gend.-overlap	991 (34)	777 (29)	890 (36)	1398 (49)	1051 (36)	532 (20)	663 (24)	1130 (38)

Means and standard errors of raw first-pass reading time (FPRT), regression-path duration (RPD) and total-fixation time (TFT) in ms and first-pass regression probability (FPRP) in percentages for each region by experimental condition. From continuous dependent variables, between-participants variance has been removed using (Cousineau, 2005)'s normalization with (Morey, 2008)'s correction factor.
