# Supplementary material for: Teasing apart retrieval and encoding interference in the processing of anaphors
Source: Front Psychol. 2015 Jun 9;6:506. doi: 10.3389/fpsyg.2015.00506 (PMC4460324; doi:10.3389/fpsyg.2015.00506)
Supplement: Supplementary file 1 [file Stimuli.pdf]

Supplementary Material: Teasing apart Retrieval and Encoding Interference in the Processing of Anaphors

Lena A. Jäger [1,*], Lena Benz [1], Jens Roeser [2], Brian W. Dillon [3] and Shravan Vasishth [1]

[1] *Department of Linguistics, University of Potsdam, Potsdam, Germany*
[2] *Department of Psychology, Nottingham Trent University, Nottingham, UK*
[3] *Department of Linguistics, University of Massachusetts, Amherst, USA*

Correspondence*:
Lena Jäger
Department of Linguistics, University of Potsdam, Karl-Liebknecht-Str. 24-25,
Potsdam, 14476, Germany, lena.jaeger@uni-potsdam.de

1 MATERIALS EXPERIMENTS 1 AND 2

1. a. Der Untertan, dem der Herrscher diktiert hat zu gehorchen, hat kurzerhand sich und die Geiseln eingemauert, mutmat der Reporter.
   Sind der Herrscher und die Geiseln eingemauert? NO
   b. Der Untertan, dem die Herrscherin diktiert hat zu gehorchen, hat kurzerhand sich und die Geiseln eingemauert, mutmat der Reporter.
   Sind die Herrscherin und die Geiseln eingemauert? NO
   c. Die Untertanin, der der Herrscher diktiert hat zu gehorchen, hat kurzerhand sich und die Geiseln eingemauert, mutmat der Reporter.
   Sind der Herrscher und die Geiseln eingemauert? NO
   d. Die Untertanin, der die Herrscherin diktiert hat zu gehorchen, hat kurzerhand sich und die Geiseln eingemauert, mutmat der Reporter.
   Sind die Herrscherin und die Geiseln eingemauert? NO
2. a. Der Gutsherr, dem der Bauer geholfen hat zu ernten, hat gütigerweise sich und das Nachbardorf ernährt, erfährt man in der Dorfchronik.
   Hat der Gutsherr genug zu essen? YES
   b. Der Gutsherr, dem die Bäuerin geholfen hat zu ernten, hat gütigerweise sich und das Nachbardorf ernährt, erfährt man in der Dorfchronik.
   Hat der Gutsherr genug zu essen? YES
   c. Die Gutsherrin, der der Bauer geholfen hat zu ernten, hat gütigerweise sich und das Nachbardorf ernährt, erfährt man in der Dorfchronik.
   Hat die Gutsherrin genug zu essen? YES
   d. Die Gutsherrin, der die Bäuerin geholfen hat zu ernten, hat gütigerweise sich und das Nachbardorf ernährt, erfährt man in der Dorfchronik.
   Hat die Gutsherrin genug zu essen? YES
3. a. Der Sohn, dem der Vater erlaubt hat zu kiffen, hat blöderweise sich und die Klassenkameraden verraten, stand im Geständnis.
   Ist der Sohn aufgeflogen? YES

b. Der Sohn, dem die Mutter erlaubt hat zu kiffen, hat blöderweise sich und die Klassenkameraden verraten, stand im Geständnis. .
Ist der Sohn aufgeflogen? YES

c. Die Tochter, der der Vater erlaubt hat zu kiffen, hat blöderweise sich und die Klassenkameraden verraten, stand im Geständnis.
Ist die Tochter aufgeflogen? YES

d. Die Tochter, der die Mutter erlaubt hat zu kiffen, hat blöderweise sich und die Klassenkameraden verraten, stand im Geständnis.
Ist die Tochter aufgeflogen? YES

4. a. Der Psychopath, dem der Krankenpfleger geholfen hat aufzustehen, hat brutalerweise sich und die Bettnachbarn gebissen, steht in der Krankenakte.
Hat der Psychopath eine Bisswunde? YES

b. Der Psychopath, dem die Krankenpflegerin geholfen hat aufzustehen, hat brutalerweise sich und die Bettnachbarn gebissen, steht in der Krankenakte.
Hat der Psychopath eine Bisswunde? YES

c. Die Psychopathin, der der Krankenpfleger geholfen hat aufzustehen, hat brutalerweise sich und die Bettnachbarn gebissen, steht in der Krankenakte.
Hat die Psychopathin eine Bisswunde? YES

d. Die Psychopathin, der die Krankenpflegerin geholfen hat aufzustehen, hat brutalerweise sich und die Bettnachbarn gebissen, steht in der Krankenakte.
Hat die Psychopathin eine Bisswunde? YES

5. a. Der Intendant, dem der Stadtrat geraten hat zurückzutreten, hat öffentlich sich und den Regisseur gelobt, schrieb das Lokalblatt.
Wurde der Intendant gelobt? YES

b. Der Intendant, dem die Stadträtin geraten hat zurückzutreten, hat öffentlich sich und den Regisseur gelobt, schrieb das Lokalblatt.
Wurde der Intendant gelobt? YES

c. Die Intendantin, der der Stadtrat geraten hat zurückzutreten, hat öffentlich sich und den Regisseur gelobt, schrieb das Lokalblatt.
Wurde die Intendantin gelobt? YES

d. Die Intendantin, der die Stadträtin geraten hat zurückzutreten, hat öffentlich sich und den Regisseur gelobt, schrieb das Lokalblatt.
Wurde die Intendantin gelobt? YES

6. a. Der Chauffeur, dem der Musikmanager angeordnet hat zu rasen, hat heimlich sich und die Diva fotografiert, zeigt das Foto auf dem Schreibtisch.
Gibt es ein Bild vom Chauffeur mit der Diva? YES

b. Der Chauffeur, dem die Musikmanagerin angeordnet hat zu rasen, hat heimlich sich und die Diva fotografiert, zeigt das Foto auf dem Schreibtisch.
Gibt es ein Bild vom Chauffeur mit der Diva? YES

c. Die Chauffeurin, der der Musikmanager angeordnet hat zu rasen, hat heimlich sich und die Diva fotografiert, zeigt das Foto auf dem Schreibtisch.
Gibt es ein Bild von der Chauffeurin mit der Diva? YES

d. Die Chauffeurin, der die Musikmanagerin angeordnet hat zu rasen, hat heimlich sich und die Diva fotografiert, zeigt das Foto auf dem Schreibtisch.
Gibt es ein Bild von der Chauffeurin mit der Diva? YES

7. a. Der Dealer, dem der Drogenabhängige versprochen hat zu bezahlen, hat grausamerweise sich und den Bewährungshelfer erschossen, mutmate die Spurensicherung.
Sind der Dealer und der Bewährungshelfer beide tot? YES

b. Der Dealer, dem die Drogenabhängige versprochen hat zu bezahlen, hat grausamerweise sich und den Bewährungshelfer erschossen, mutmate die Spurensicherung.
Sind der Dealer und der Bewährungshelfer beide tot? YES

c. Die Dealerin, der der Drogenabhängige versprochen hat zu bezahlen, hat grausamerweise sich und den Bewährungshelfer erschossen, mutmate die Spurensicherung.
Sind die Dealerin und der Bewährungshelfer beide tot? YES

d. Die Dealerin, der die Drogenabhängige versprochen hat zu bezahlen, hat grausamerweise sich und den Bewährungshelfer erschossen, mutmate die Spurensicherung.
Sind die Dealerin und der Bewährungshelfer beide tot? YES

8. a. Der Zauberer, dem der Prinz gelobt hat gerecht zu herrschen, hat schlielich sich und das Königreich gerettet, erzählte der Grovater.
Sind der Zauberer und das Königreich gerettet? YES

b. Der Zauberer, dem die Prinzessin gelobt hat gerecht zu herrschen, hat schlielich sich und das Königreich gerettet, erzählte der Grovater.
Sind der Zauberer und das Königreich gerettet? YES

c. Die Zauberin, der der Prinz gelobt hat gerecht zu herrschen, hat schlielich sich und das Königreich gerettet, erzählte der Grovater. ? Sind die Zauberin und das Königreich gerettet? YES

d. Die Zauberin, der die Prinzessin gelobt hat gerecht zu herrschen, hat schlielich sich und das Königreich gerettet, erzählte der Grovater.
Sind die Zauberin und das Königreich gerettet? YES

9. a. Der Magier, dem der Veranstalter geschworen hat zu schweigen, hat gestern sich und das Publikum verhext, behauptet Hänschen.
Waren gestern Magier und Publikum verhext? YES

b. Der Magier, dem die Veranstalterin geschworen hat zu schweigen, hat gestern sich und das Publikum verhext, behauptet Hänschen.
Waren gestern Magier und Publikum verhext? YES

c. Die Magierin, der der Veranstalter geschworen hat zu schweigen, hat gestern sich und das Publikum verhext, behauptet Hänschen.
Waren gestern Magierin und Publikum verhext? YES

d. Die Magierin, der die Veranstalterin geschworen hat zu schweigen, hat gestern sich und das Publikum verhext, behauptet Hänschen.
Waren gestern Magierin und Publikum verhext? YES

10. a. Der Soldat, dem der Offizier gestattet hat zu schieen, hat versehentlich sich und die Kameraden verletzt, heit es im Bericht an den Verteidigungsminister.
Ist der Offizier verletzt? NO

b. Der Soldat, dem die Offizierin gestattet hat zu schieen, hat versehentlich sich und die Kameraden verletzt, heit es im Bericht an den Verteidigungsminister.
Ist die Offizierin verletzt? NO

c. Die Soldatin, der der Offizier gestattet hat zu schieen, hat versehentlich sich und die Kameraden verletzt, heit es im Bericht an den Verteidigungsminister.
Ist der Offizier verletzt? NO

d. Die Soldatin, der die Offizierin gestattet hat zu schieen, hat versehentlich sich und die Kameraden verletzt, heit es im Bericht an den Verteidigungsminister.
Ist die Offizierin verletzt? NO

11. a. Der Autofahrer, dem der Verkehrspolizist verboten hat weiterzufahren, hat nachweislich sich und den Beifahrer gefährdet, steht in der Akte.
War der Verkehrspolizist in Gefahr? NO

b. Der Autofahrer, dem die Verkehrspolizistin verboten hat weiterzufahren, hat nachweislich sich und den Beifahrer gefährdet, steht in der Akte.
War die Verkehrspolizistin in Gefahr? NO

c. Die Autofahrerin, der der Verkehrspolizist verboten hat weiterzufahren, hat nachweislich sich und den Beifahrer gefährdet, steht in der Akte.
War der Verkehrspolizist in Gefahr? NO

d. Die Autofahrerin, der die Verkehrspolizistin verboten hat weiterzufahren, hat nachweislich sich und den Beifahrer gefährdet, steht in der Akte.
War die Verkehrspolizistin in Gefahr? NO

12. a. Der Dieb, dem der Hehler befohlen hat zu stehlen, hat überraschenderweise sich und die Kollegen angezeigt, berichtete das Hochglanzmagazin.
Hat der Hehler eine Anzeige? NO

b. Der Dieb, dem die Hehlerin befohlen hat zu stehlen, hat überraschenderweise sich und die Kollegen angezeigt, berichtete das Hochglanzmagazin.
Hat die Hehlerin eine Anzeige? NO

c. Die Diebin, der der Hehler befohlen hat zu stehlen, hat überraschenderweise sich und die Kollegen angezeigt, berichtete das Hochglanzmagazin.
Hat der Hehler eine Anzeige? NO

d. Die Diebin, der die Hehlerin befohlen hat zu stehlen, hat überraschenderweise sich und die Kollegen angezeigt, berichtete das Hochglanzmagazin.
Hat die Hehlerin eine Anzeige? NO

13. a. Der Schüler, dem der Vater empfohlen hat zu schleimen, hat dummerweise sich und die Klasse blamiert, steht in der Schülerzeitung.
Erlitt der Vater die Blamage? NO

b. Der Schüler, dem die Mutter empfohlen hat zu schleimen, hat dummerweise sich und die Klasse blamiert, steht in der Schülerzeitung.
Erlitt die Mutter die Blamage? NO

c. Die Schülerin, der der Vater empfohlen hat zu schleimen, hat dummerweise sich und die Klasse blamiert, steht in der Schülerzeitung.
Erlitt der Vater die Blamage? NO

d. Die Schülerin, der die Mutter empfohlen hat zu schleimen, hat dummerweise sich und die Klasse blamiert, steht in der Schülerzeitung.
Erlitt die Mutter die Blamage? NO

14. a. Der Patient, dem der Arzt vorgeschlagen hat zu fasten, hat lustigerweise sich und die Krankenschwester bekocht, glaubt das Pflegepersonal.
Haben der Arzt und die Krankenschwester gemeinsam gegessen? NO

b. Der Patient, dem die Ärztin vorgeschlagen hat zu fasten, hat lustigerweise sich und die Krankenschwester bekocht, glaubt das Pflegepersonal.
Haben die Ärztin und die Krankenschwester gemeinsam gegessen? NO

c. Die Patientin, der der Arzt vorgeschlagen hat zu fasten, hat lustigerweise sich und die Krankenschwester bekocht, glaubt das Pflegepersonal.
Haben der Arzt und die Krankenschwester gemeinsam gegessen? NO

d. Die Patientin, der die Ärztin vorgeschlagen hat zu fasten, hat lustigerweise sich und die Krankenschwester bekocht, glaubt das Pflegepersonal.
Haben die Ärztin und die Krankenschwester gemeinsam gegessen? NO

15. a. Der Bettler, dem der Millionär ermöglicht hat zu reisen, hat absichtlich sich und den Matrosen ermordet, bemerkt der Kapitän.
Sind der Millionär und der Matrose beide tot? NO

b. Der Bettler, dem die Millionärin ermöglicht hat zu reisen, hat absichtlich sich und den Matrosen ermordet, bemerkt der Kapitän.
Sind die Millionärin und der Matrose beide tot? NO
c. Die Bettlerin, der der Millionär ermöglicht hat zu reisen, hat absichtlich sich und den Matrosen ermordet, bemerkt der Kapitän.
Sind der Millionär und der Matrose beide tot? NO
d. Die Bettlerin, der die Millionärin ermöglicht hat zu reisen, hat absichtlich sich und den Matrosen ermordet, bemerkt der Kapitän.
Sind die Millionärin und der Matrose beide tot? NO

16. a. Der Arbeitslose, dem der Beamte auferlegt hat zu arbeiten, hat schrecklicherweise sich und die Kinder vergiftet, schreibt die Bild.
Sind der Beamte und die Kinder vergiftet? NO
b. Der Arbeitslose, dem die Beamtin auferlegt hat zu arbeiten, hat schrecklicherweise sich und die Kinder vergiftet, schreibt die Bild.
Sind die Beamtin und die Kinder vergiftet? NO
c. Die Arbeitslose, der der Beamte auferlegt hat zu arbeiten, hat schrecklicherweise sich und die Kinder vergiftet, schreibt die Bild.
Sind der Beamte und die Kinder vergiftet? NO
d. Die Arbeitslose, der die Beamtin auferlegt hat zu arbeiten, hat schrecklicherweise sich und die Kinder vergiftet, schreibt die Bild.
Sind die Beamtin und die Kinder vergiftet? NO

2 MATERIALS EXPERIMENT 3

1. a. Åke säger att Alf jobbade med hans sysslingar på helgerna.
Jobbade Alf med Åkes sysslingar? YES
b. Åke säger att Ann jobbade med hans sysslingar på helgerna.
Jobbade Ann med Åkes sysslingar? YES
c. Åke som Alf tackade ringer sina sysslingar på kvällen.
Har Åke sysslingar? YES
d. Åke som Ann tackade ringer sina sysslingar på kvällen.
Har Åke sysslingar? YES

2. a. Åsa skriver att Eva dödade hennes patienter med fel behandling.
Dödade Eva Åsas patienter? YES
b. Åsa skriver att Dan dödade hennes patienter med fel behandling.
Dödade Dan Åsas patienter? YES
c. Åsa som Eva kände talar med sina patienter på sjukhuset.
Har Åsa patienter som hon talar med? YES
d. Åsa som Dan kände talar med sina patienter på sjukhuset.
Har Åsa patienter som hon talar med? YES

3. a. Jan anser att Max testade hans sjukdomar genom ett blodprov.
Testade Max Jans sjukdomar? YES
b. Jan anser att Gun testade hans sjukdomar genom ett blodprov.
Testade Gun Jans sjukdomar? YES

c. Jan som Max beundrade glömde sina uppgifter till nästa vecka.
Har Jan uppgifter som han glömde? YES

d. Jan som Gun beundrade glömde sina uppgifter till nästa vecka.
Har Jan uppgifter som han glömde? YES

4. a. Ida menar att Moa beundrade hennes teorier om kvantfysik.
Beundrade Moa Idas teorier? YES

b. Ida menar att Ola beundrade hennes teorier om kvantfysik.
Beundrade Ola Idas teorier? YES

c. Ida som Moa frågade väntar på sina föräldrar vid ingången.
Är det Idas föräldrar hon väntar på? YES

d. Ida som Ola frågade väntar på sina föräldrar vid ingången.
Är det Idas föräldrar hon väntar på? YES

5. a. Ove meddelar att Per tvingade hans studenter att sjunga.
Tvingade Per Oves studenter att sjunga? YES

b. Ove meddelar att Pia tvingade hans studenter att sjunga.
Tvingade Pia Oves studenter att sjunga? YES

c. Ove som Per slog citerar sina texter på varje sida.
Är det Oves texter? YES

d. Ove som Pia slog citerar sina texter på varje sida.
Är det Oves texter? YES

6. a. Rut noterar att Siv besökte hennes mostrar vid jul.
Besökte Siv Ruts mostrar? YES

b. Rut noterar att Ulf besökte hennes mostrar vid jul.
Besökte Ulf Ruts mostrar? YES

c. Rut som Siv ringde älskar sina döttrar lika mycket.
Har Rut döttrar som hon älskar? YES

d. Rut som Ulf ringde älskar sina döttrar lika mycket.
Har Rut döttrar som hon älskar? YES

7. a. Adam nämner att Arne förändrade hans uppfattningar genom den boken.
Förändrade Arne Adams uppfattningar? YES

b. Adam nämner att Anna förändrade hans uppfattningar genom den boken.
Förändrade Anna Adams uppfattningar? YES

c. Adam som Arne hjälpte lyssnar på sina kompisar på konserten.
Är det Adams kompisar? YES

d. Adam som Anna hjälpte lyssnar på sina kompisar på konserten.
Är det Adams kompisar? YES

8. a. Ebba inser att Elin röstade på hennes fiender av strategiska skäl.
Röstade Elin på Ebbas fiender? YES

b. Ebba inser att Axel röstade på hennes fiender av strategiska skäl.
Röstade Axel på Ebbas fiender? YES

c. Ebba som Elin betalade betraktar sina tolkningar av de sista uppgifterna.
Är det Ebbas tolkningar? YES

d. Ebba som Axel betalade betraktar sina tolkningar av de sista uppgifterna.
Är det Ebbas tolkningar? YES

9. a. Emil bevisade att Erik ändrade hans referenser på Wikipedia.
Ändrade Erik referenserna på Wikipedia? YES

b. Emil bevisade att Ella ändrade hans referenser på Wikipedia.
Ändrade Ella referenserna på Wikipedia? YES
c. Emil som Erik älskade ändrar sina åsikter om politik.
Älskade Erik Emil? YES
d. Emil som Ella älskade ändrar sina åsikter om politik.
Älskade Ella Emil? YES

10. a. Elsa förstår att Emma saknar hennes hundar sedan igår.
Saknar Emma hundarna? YES
b. Elsa förstår att Hans saknar hennes hundar sedan igår.
Saknar Hans hundarna YES
c. Elsa som Emma saknar försvarar sina ider med stolthet.
Saknar Emma Elsa? YES
d. Elsa som Hans saknar försvarar sina ider med stolthet.
Saknar Hans Elsa? YES

11. a. Hugo hoppas att John betalade hans döttrar för hjälpen.
Hjälpte döttrarna John? YES
b. Hugo hoppas att Gerd betalade hans döttrar för hjälpen.
Hjälpte döttrarna Gerd? YES
c. Hugo som John betraktade gillar sina chefer sedan förra mötet.
Betraktade John Hugo? YES
d. Hugo som Gerd betraktade gillar sina chefer sedan förra mötet.
Betraktade Gerd Hugo? YES

12. a. Inga glömmer att Lena räddade hennes katter undan hunden.
Räddade Lena katterna? YES
b. Inga glömmer att Karl räddade hennes katter undan hunden.
Räddade Karl katterna? YES
c. Inga som Lena sökte frågar sina studenter om dagens läxa.
Sökte Lena Inga? YES
d. Inga som Karl sökte frågar sina studenter om dagens läxa.
Sökte Karl Inga? YES

13. a. Lars svarar att Leif hjälpte hans föräldrar när de flyttade.
Hjälpte Leif Lars föräldrar? YES
b. Lars svarar att Lina hjälpte hans föräldrar när de flyttade.
Hjälpte Lina Lars föräldrar? YES
c. Lars som Leif hittade stryker sina hundar över öronen.
Har Lars hundar? YES
d. Lars som Lina hittade stryker sina hundar över öronen.
Har Lars hundar? YES

14. a. Lisa försvarar att Maja uppmanade hennes elever att äta upp.
Uppmanade Maja Lisas elever? YES
b. Lisa försvarar att Mats uppmanade hennes elever att äta upp.
Uppmanade Mats Lisas elever? YES
c. Lisa som Maja gillade tittar på sina planer för bröllopet.
Har Lisa planer för bröllopet? YES
d. Lisa som Mats gillade tittar på sina planer för bröllopet.
Har Lisa planer för bröllopet? YES

15. a. Olof visste att Sten ersatte hans dörrar efter festen.
   Ersatte Sten Olofs dörrar? YES
   b. Olof visste att Mona ersatte hans dörrar efter festen.
   Ersatte Mona Olofs dörrar? YES
   c. Olof som Sten svarade arbetar med sina datorer till projektet.
   Är det Olofs datorer? YES
   d. Olof som Mona svarade arbetar med sina datorer till projektet.
   Är det Olofs datorer? YES
16. a. Sara stödjer att Ulla vaccinerade hennes hästar innan epidemin.
   Vaccinerade Ulla Saras hästar? YES
   b. Sara stödjer att Sven vaccinerade hennes hästar innan epidemin.
   Vaccinerade Sven Saras hästar? YES
   c. Sara som Ulla besökte berömmer sina kollegor under mötet.
   Är det Saras kollegor? YES
   d. Sara som Sven besökte berömmer sina kollegor under mötet.
   Är det Saras kollegor? YES
17. a. Albin redovisar att Allan pratade med hans chefer om semester.
   Pratade Allan med Albins chefer? YES
   b. Albin redovisar att Alice pratade med hans chefer om semester.
   Pratade Alice med Albins chefer? YES
   c. Albin som Allan talade med spelar med sina kompisar på skollovet.
   Har Albin kompisar? YES
   d. Albin som Alice talade med spelar med sina kompisar på skollovet.
   Har Albin kompisar? YES
18. a. Anita bekräftar att Berit hotade hennes reformer om arbetslöshet.
   Hotade Berit Anitas reformer? YES
   b. Anita bekräftar att Anton hotade hennes reformer om arbetslöshet.
   Hotade Anton Anitas reformer? YES
   c. Anita som Berit pratade med stödjer sina reformer om naturskydd.
   Är det Anitas reformer? YES
   d. Anita som Anton pratade med stödjer sina reformer om naturskydd.
   Är det Anitas reformer? YES
19. a. Arvid uppskattar att Bengt upptäckte hans skador på benet.
   Upptäckte Bengt Arvids skador? YES
   b. Arvid uppskattar att Britt upptäckte hans skador på benet.
   Upptäckte Britt Arvids skador? YES
   c. Arvid som Bengt kollade undersöker sina skador efter olyckan.
   Är Arvid skadad? YES
   d. Arvid som Britt kollade undersöker sina skador efter olyckan.
   Är Arvid skadad? YES
20. a. Ellen misstänker att Erika matade hennes barn den sista natten.
   Matade Erika Ellens barn? YES
   b. Ellen misstänker att Bernt matade hennes barn den sista natten.
   Matade Bernt Ellens barn? YES
   c. Ellen som Erika arbetade med träffar sina kunder på konferensen.
   Är det Ellens kunder? YES

d. Ellen som Bernt arbetade med träffar sina kunder på konferensen.
Är det Ellens kunder? YES

21. a. Björn befarade att David nämnde hans rapporter på hemsidan.
Befarade Björn att nämnas på hemsidan? YES
b. Björn befarade att Frida nämnde hans rapporter på hemsidan.
Befarade Björn att nämnas på hemsidan? YES
c. Björn som David försvarade utvärderade sina metoder efter utredningen.
Försvarade David Björn? YES
d. Björn som Frida försvarade utvärderade sina metoder efter utredningen.
Försvarade Frida Björn? YES

22. a. Hanna förbjuder att Helen tackar hennes lärare för undervisningen.
Förbjuder Hanna att tacka lärarna? YES
b. Hanna förbjuder att Elias tackar hennes lärare för undervisningen.
Förbjuder Hanna att tacka lärarna? YES
c. Hanna som Helen räddade berättar om sina upplevelser från katastrofen.
Är upplevelserna från katastrofen? YES
d. Hanna som Elias räddade berättar om sina upplevelser från katastrofen.
Är upplevelserna från katastrofen? YES

23. a. Filip hindrar att Georg motbevisar hans tolkningar av boken.
Hindrar Filip Georg att motbevisa tolkningarna? YES
b. Filip hindrar att Inger motbevisar hans tolkningar av boken.
Hindrar Filip Georg att motbevisa tolkningarna? YES
c. Filip som Georg träffade frågar sina arbetare att komma tillbaka.
Frågar Filip arbetarna? YES
d. Filip som Inger träffade frågar sina arbetare att komma tillbaka.
Frågar Filip arbetarna? YES

24. a. Irene tillåter att Jenny kollar på hennes bilder från semestern.
Kollade Jenny på bilderna från semestern? YES
b. Irene tillåter att Håkan kollar på hennes bilder från semestern.
Kollade Jenny bilderna från semestern? YES
c. Irene som Jenny kontrollerade leder sina trupper mot slagfältet.
Kontrollerade Jenny Irene? YES
d. Irene som Håkan kontrollerade leder sina trupper mot slagfältet.
Kontrollerade Håkan Irene? YES

25. a. Jakob berättar att Jimmy väckte hans hyresgäster vid branden.
Väckte Jakob Jimmys hyresgäster? NO
b. Jakob berättar att Julia väckte hans hyresgäster vid branden.
Väckte Jakob Julias hyresgäster? NO
c. Jakob som Jimmy spelade med varnade sina hyresgäster på grund av branden.
Är det Jimmys hyresgäster? NO
d. Jakob som Julia spelade med varnade sina hyresgäster på grund av branden.
Är det Julias hyresgäster? NO

26. a. Karin föreslår att Klara träffar hennes vänner på middag.
Är förslaget att Karin träffar Klaras vänner? NO
b. Karin föreslår att Johan träffar hennes vänner på middag.
Är förslaget att Karin träffar Johans vänner? NO

c. Karin som Klara skyddade dödar sina arbetskamrater på grund av hämnd.
Var det Klaras arbetskamrater? NO

d. Karin som Johan skyddade dödar sina arbetskamrater på grund av hämnd.
Var det Johans arbetskamrater? NO

27. a. Jonas hävdar att Kjell rörde hans ringar med avsikt.
Är det Kjells ringar? NO

b. Jonas hävdar att Linda rörde hans ringar med avsikt.
Är det Lindas ringar? NO

c. Jonas som Kjell jobbade med beundrar sina artiklar om Nobelpriset.
Är det Kjells artiklar? NO

d. Jonas som Linda jobbade med beundrar sina artiklar om Nobelpriset.
Är det Lindas artiklar? NO

28. a. Malin förstod att Maria sökte hennes lägenheter för pengarna.
Är det Marias lägenheter? NO

b. Malin förstod att Linus sökte hennes lägenheter för pengarna.
Är det Linus lägenheter? NO

c. Malin som Maria tittade på testar sina råttor i laboratoriet.
Är det Marias råttor? NO

d. Malin som Linus tittade på testar sina råttor i laboratoriet.
Är det Linus råttor? NO

29. a. Lucas meddelar att Oskar stödde hans trupper under upproret.
Är det Oskars trupper? NO

b. Lucas meddelar att Marie stödde hans trupper under upproret.
Är det Maries trupper? NO

c. Lucas som Oskar väntade på nämnde sina vinster i lotteriet.
Är det Oskars vinster? NO

d. Lucas som Marie väntade på nämnde sina vinster i lotteriet.
Är det Maries vinster? NO

30. a. Märta upptäcker att Sofia läser hennes studier om sömnstörningar.
Läser Märta Sofias studier? NO

b. Märta upptäcker att Peter läser hennes studier om sömnstörningar.
Läser Märta Peters studier? NO

c. Märta som Sofia upptäckte betalar sina filmer med pengar.
Är det Sofias filmer? NO

d. Märta som Peter upptäckte betalar sina filmer med pengar.
Är det Peters filmer? NO

31. a. Roger tror att Simon förstörde hans filmer med överflödiga effekter.
Är det Simons filmer? NO

b. Roger tror att Sonja förstörde hans filmer med överflödiga effekter.
Är det Sonjas filmer? NO

c. Roger som Simon väckte räddar sina modeller från hyllan.
Räddar Roger Simons modeller? NO

d. Roger som Sonja väckte räddar sina modeller från hyllan.
Räddar Roger Sonjas modeller? NO

32. a. Viola tycker att Wilma betraktade hennes resultat med skepsis.
    Är det Wilmas resultat? NO
    b. Viola tycker att Tommy betraktade hennes resultat med skepsis.
    Är det Tommys resultat? NO
    c. Viola som Wilma citerade tackar sina ministrar för korrigeringarna.
    Tackar Viola Wilmas ministrar? NO
    d. Viola som Tommy citerade tackar sina ministrar för korrigeringarna.
    Tackar Viola Tommys ministrar? NO
33. a. Anders tänker att Daniel gillade hans samlingar med gamla viner.
    Samlar Anders gamla bilar? NO
    b. Anders tänker att Agneta gillade hans samlingar med gamla viner.
    Samlar Anders gamla bilar? NO
    c. Anders som Daniel respekterade hotar sina grannar på campingen.
    Hotar Daniel grannarna? NO
    d. Anders som Agneta respekterade hotar sina grannar på campingen.
    Hotar Agneta grannarna? NO
34. a. Amanda misstänker att Anette talade med hennes ministrar om nedrustning.
    Talade Amanda med ministrarna om miljöskydd? NO
    b. Amanda misstänker att Gustav talade med hennes ministrar om nedrustning.
    Talade Amanda med ministrarna om miljöskydd? NO
    c. Amanda som Anette förmanade förändrar sina känslor om kungen.
    Förmanade Amanda Anette? NO
    d. Amanda som Gustav förmanade förändrar sina känslor om kungen.
    Förmanade Amanda Gustav? NO
35. a. Henrik anger att Jesper citerade hans intervjuer i rapporten.
    Citerade Henrik intervjuerna? NO
    b. Henrik anger att Anneli citerade hans intervjuer i rapporten.
    Citerade Henrik intervjuerna? NO
    c. Henrik som Jesper nämnde pratar med sina barn om resan.
    Pratade Henrik med vännerna om resan? NO
    d. Henrik som Anneli nämnde pratar med sina barn om resan.
    Pratade Henrik med vännerna om resan? NO
36. a. Annika bedömer att Astrid försvarade hennes ändringar av inredningen.
    Försvarade Annika ändringarna? NO
    b. Annika bedömer att Joakim försvarade hennes ändringar av inredningen.
    Försvarade Annika ändringarna? NO
    c. Annika som Astrid ersatte kollar sina ändringar i artiklarna.
    Ersatte Annika Astrid? NO
    d. Annika som Joakim ersatte kollar sina ändringar i artiklarna.
    Ersatte Annika Joakim? NO
37. a. Jörgen märker att Ludvig älskade hans bloggar om vetenskap.
    Älskade Jörgen Ludvigs bloggar? NO
    b. Jörgen märker att Birgit älskade hans bloggar om vetenskap.
    Älskade Jörgen Birgits bloggar? NO
    c. Jörgen som Ludvig besegrade uppmanar sina läkare att vara försiktiga.
    Är det Ludvigs läkare? NO

d. Jörgen som Birgit besegrade uppmanar sina läkare att vara försiktiga.
Är det Birgits läkare? NO

38. a. Britta uppmanar att Carina frågar hennes läkare om hjälp.
Är det Carinas läkare? NO
b. Britta uppmanar att Magnus frågar hennes läkare om hjälp.
Är det Magnus läkare? NO
c. Britta som Carina anställde förlorade sina vänner på det gamla slottet.
Förlorade Britta Carinas vänner? NO
d. Britta som Magnus anställde förlorade sina vänner på det gamla slottet.
Förlorade Britta Magnus vänner? NO

39. a. Marcus avslöjar att Martin stödde hans medlemmar genom donationer.
Stödde Marcus Martins medlemmar? NO
b. Marcus avslöjar att Emilia stödde hans medlemmar genom donationer.
Stödde Marcus Emilias medlemmar? NO
c. Marcus som Martin skrev om besöker sina lägenheter i Italien.
Har Martin lägenheter i Italien? NO
d. Marcus som Emilia skrev om besöker sina lägenheter i Italien.
Har Emilia lägenheter i Italien? NO

40. a. Gunnel krävde att Helena skulle höra hennes nyheter om praktikplatsen.
Skulle Gunnel höra om Helenas praktikplats? NO
b. Gunnel krävde att Mikael skulle höra hennes nyheter om praktikplatsen.
Skulle Gunnel höra om Mikaels praktikplats? NO
c. Gunnel som Helena lyssnade på hittar sina pengar vid skrivbordet.
Hittade hon Helenas pengar vid skrivbordet? NO
d. Gunnel som Mikael lyssnade på hittar sina pengar vid skrivbordet.
Hittade hon Mikaels pengar vid skrivbordet? NO

41. a. Niklas gillar att Oliver väntade på hans tidningar på kontoret.
Väntade Niklas på Olivers tidningar? NO
b. Niklas gillar att Ingela väntade på hans tidningar på kontoret.
Väntade Niklas på Ingelas tidningar? NO
c. Niklas som Oliver röstade på upptäcker sina fördelar med pensionen.
Upptäcker Niklas Olivers fördelar? NO
d. Niklas som Ingela röstade på upptäcker sina fördelar med pensionen.
Upptäcker Niklas Ingelas fördelar? NO

42. a. Ingrid erkänner att Linna uppmuntrade hennes morbröder under tävlingen.
Uppmuntrade Ingrid Linnas morbröder? NO
b. Ingrid erkänner att Patrik uppmuntrade hennes morbröder under tävlingen.
Uppmuntrade Ingrid Patriks morbröder? NO
c. Ingrid som Linna aktade jobbar med sina verk i femton år.
Jobbar Ingrid med Linnas verk i femton år? NO
d. Ingrid som Patrik aktade jobbar med sina verk i femton år.
Jobbar Ingrid med Patriks verk i femton år? NO

43. a. Rasmus godkänner att Robert skojade med hans kunder i klagomålet.
Skojade Rasmus med Roberts kunder? NO
b. Rasmus godkänner att Louise skojade med hans kunder i klagomålet.
Skojade Rasmus med Louises kunder? NO

c. Rasmus som Robert beundrar hjälper sina klienter i rättegången.
Hjälper Rasmus Roberts klienter? NO

d. Rasmus som Louise beundrar hjälper sina klienter i rättegången.
Hjälper Rasmus Louises klienter? NO

44. a. Lovisa beslutar att Monica måste ta hand om hennes växter i vinterträdgården.
Måste Lovisa ta hand om Monicas växter? NO

b. Lovisa beslutar att Stefan måste ta hand om hennes växter i vinterträdgården.
Måste Lovisa ta hand om Stefans växter? NO

c. Lovisa som Monica hotade ger sina hästar dålig mat.
Gav Lovisa Monicas hästar dålig mat? NO

d. Lovisa som Stefan hotade ger sina hästar dålig mat.
Gav Lovisa Stefans hästar dålig mat? NO

45. a. Thomas känner att Tobias försvarade hans principer i domstolen.
Känner Thomas att Tobias försvarade principerna i hushållet? NO

b. Thomas känner att Sandra försvarade hans principer i domstolen.
Känner Thomas att Sandra försvarade principerna i hushållet? NO

c. Thomas som Tobias förändrade bedrar sina investerare med oväntade utgifter.
Förändrade Thomas Tobias? NO

d. Thomas som Sandra förändrade bedrar sina investerare med oväntade utgifter.
Förändrade Thomas Sandra? NO

46. a. Ulrika hävdade att Yvonne letar efter hennes miljoner på fel ställe.
Letar Ulrika efter miljonerna? NO

b. Ulrika hävdade att Viktor letar efter hennes miljoner på fel ställe.
Letar Ulrika efter miljonerna? NO

c. Ulrika som Yvonne tvingade nämner sina ledamöter i undersökningen.
Tvingade Ulrika Yvonne? NO

d. Ulrika som Viktor tvingade nämner sina ledamöter i undersökningen.
Tvingade Ulrika Viktor? NO

47. a. Andreas rapporterade att Emanuel lyssnade på hans berättelser för inspiration.
Lyssnade Emanuel på musik för inspiration? NO

b. Andreas rapporterade att Camilla lyssnade på hans berättelser för inspiration.
Lyssnade Camilla på musik för inspiration? NO

c. Andreas som Emanuel stödde förstummade sina väljare med utopiska uppfattningar.
Stödde Andreas Emanuel? NO

d. Andreas som Camilla stödde förstummade sina väljare med utopiska uppfattningar.
Stödde Andreas Camilla? NO

48. a. Cecilia minns att Jessica ringde hennes ledamöter innan beslutet.
Ringde Cecilia sina läkarna innan beslutet? NO

b. Cecilia minns att Fredrik ringde hennes ledamöter innan beslutet.
Ringde Cecilia sina läkarna innan beslutet? NO

c. Cecilia som Jessica testade svarade sina fiender med osäkerhet.
Svarade Cecilia med övertygelse? NO

d. Cecilia som Fredrik testade svarade sina fiender med osäkerhet.
Svarade Cecilia med övertygelse? NO
